# The pes of *Australovenator wintonensis* (Theropoda: Megaraptoridae): analysis of the pedal range of motion and biological restoration

**DOI:** 10.7717/peerj.2312

**Published:** 2016-08-03

**Authors:** Matt A. White, Alex G. Cook, Ada J. Klinkhamer, David A. Elliott

**Affiliations:** 1 School of Engineering, University of Newcastle, Callaghan, New South Wales, Australia; 2 Palaeontology, Australian Age of Dinosaurs Museum of Natural History, Winton, Queensland, Australia; 3 School of Earth Sciences, University of Queensland, St Lucia, Queensland, Australia; 4 School of Environmental and Rural Sciences, University of New England, Armidale, New South Wales, Australia

**Keywords:** Australovenator, Theropod, Pes, Reconstruction, Range of motion, Dinosaurs, Foot print

## Abstract

The pedal range of motion in *Australovenator wintonensis* is investigated to determine what influence soft tissue had on range of motion in the foot. Fortunately, the theropod pes shares a close morphology with extant large cursorial birds. Therefore, to better understand the pedal range of motion of *Australovenator*, the pedal range of motion of *Dromaius novaehollandiae* (commonly known as the emu) was analysed with and without soft tissue. We used a variety of innovative digital techniques to analyse the range of motion and biologically restore the *Australovenator* pes. Computed tomography scans of *Dromaius* pes in fully flexed and fully extended positions provided the soft tissue range of motion limits. The bone on bone range of motion of the same specimen was replicated following the removal of soft tissue. It was identified that there was an increase in range of motion potential with the removal of soft tissue. This variation provided a guide to develop the potential range of motion of a fully fleshed *Australovenator* pes. Additionally, the dissection of the *Dromaius* pes provided a guide enabling the replication of the corresponding soft tissue and keratin sheaths of the *Australovenator* pes.

## Introduction

**Figure 1:**
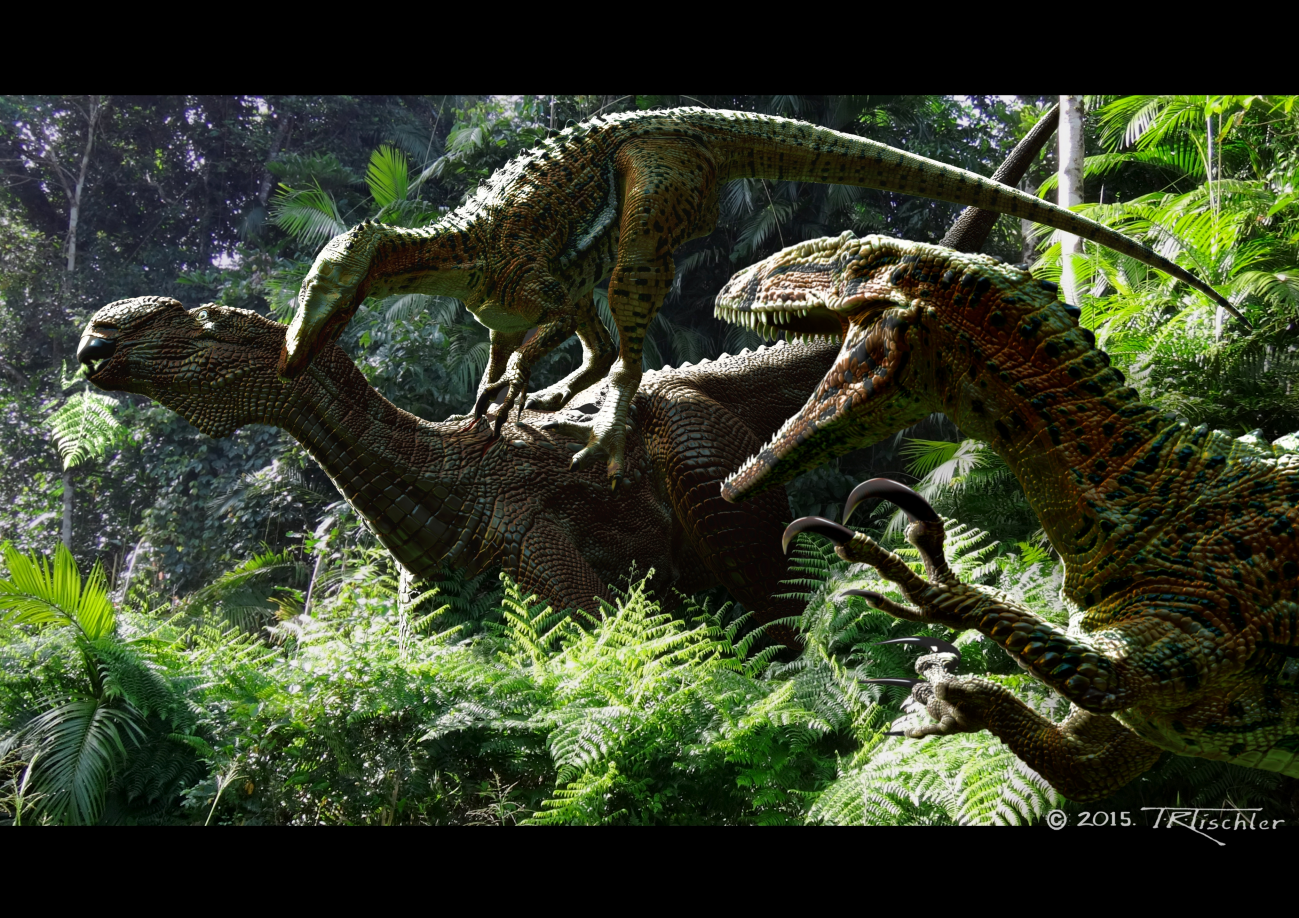
Australovenator wintonensis. *Australovenator* attacking a *Muttaburrasaurus*. Artwork by Travis R. Tischler.

**Figure 2:**
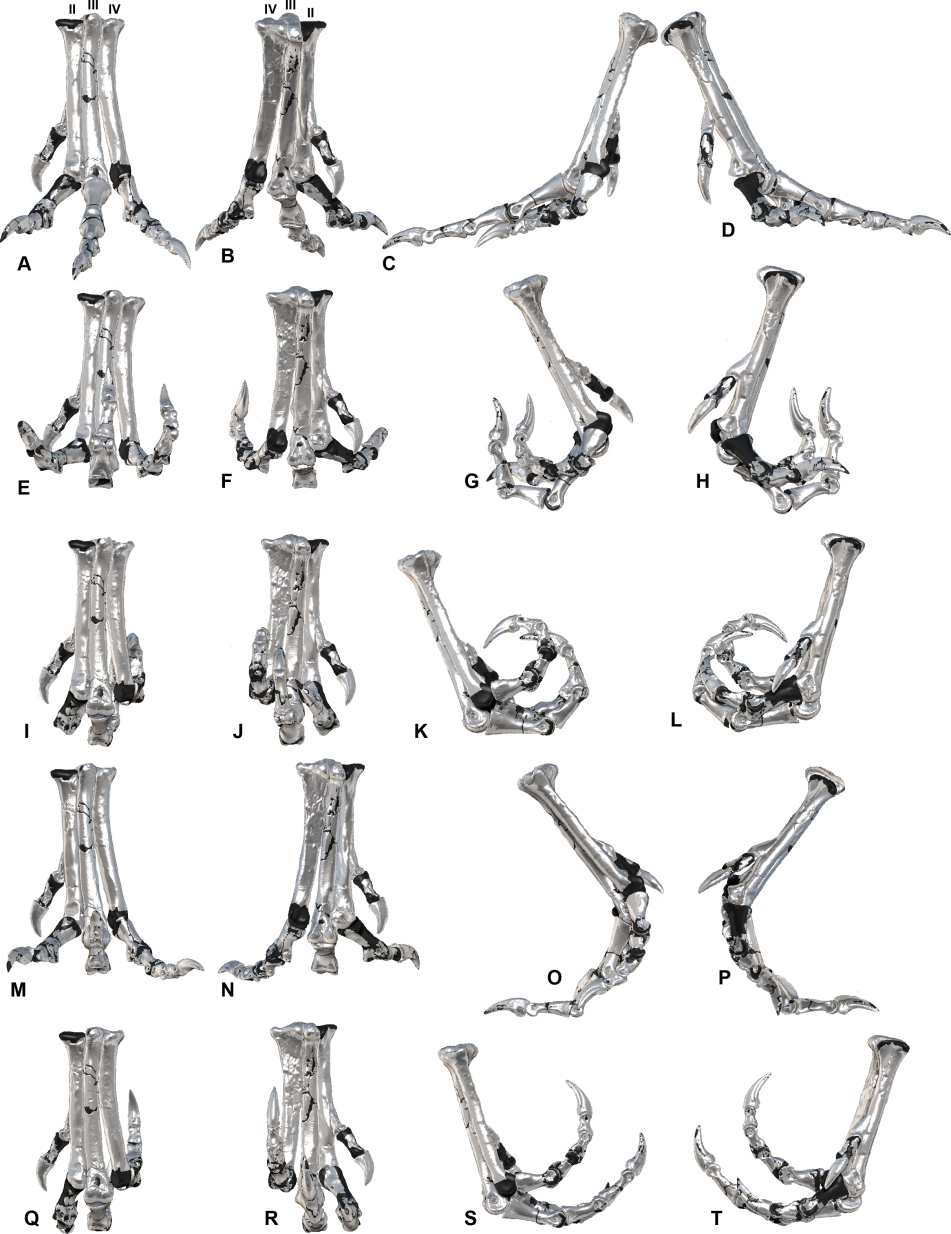
ROM of the *Australovenator* pedal digits with and without soft tissue. The black coloration represents missing elements. Neutral posture: (A) Anterior; (B) Plantar; (C) Lateral; (D) Medial. Extended without soft tissue: (E) Anterior; (F) Plantar; (G) Lateral; (H) Medial. Flexed without soft tissue: (I) Anterior; (J) Plantar; (K) Lateral; (L) Medial. Extended with soft tissue: (M) Anterior; (N) Plantar; (O) Lateral; (P) Medial. Flexed with soft tissue: (Q) Anterior; (R) Plantar; (S) Lateral; (T) Medial.

*Australovenator wintonensis* Hocknull et al., 2009 is a Megaraptorid theropod discovered in Cenomanian aged deposits of the Winton Formation in Central Queensland, Australia (Bryan et al., 2012; White et al., 2013b) (Fig. 1). It was discovered by the Australian Age of Dinosaur Museum of Natural History in 2006 and formally described in 2009 (Hocknull et al., 2009). Since its description additional skeletal elements have been reported in White et al. (2012), White et al. (2013a) and White et al. (2015b). More recent elements include a pedal ungual III-4 and a pedal phalanx I-1. The discovery of pedal phalanx I-1 completed the *Australoventor* pedal arcade, which prompted a revision of the pedal phalanges. It was identified that some of the specimens were reported in the incorrect positions (Hocknull et al., 2009; White et al., 2012), which are here rectified. With the complete pes now known and articulated, individual specimens can be properly restored and their corresponding range of motion (ROM) determined. The ROM limits were determined by orientating the metatarsus at maximum extension with phalanx III-1 horizontal as in walking birds immediately before the foot is lifted (Senter, 2009). The neutral position (0°) represents the horizontal articulation with the buttressing phalanx (Fig. 2). The ROM of the joint between pedal phalanges II-2 and II-3 of *Australovenator* was difficult to determine due to an abnormality on the proximal end of the ungual.

Soft tissue has the potential to play a major role in determining the accuracy of results in ROM reconstructions of extinct archosaurs. Therefore it was decided to not only look at typical bone on bone analysis (Carpenter, 2002; Carpenter & Smith, 2001; Galton, 1971; Kobayashi & Barsbold, 2005; Senter, 2005; Senter & Robins, 2005; Senter, 2009; White et al., 2015a) but also investigate how soft tissue influences ROM. Soft tissues have been investigated previously to determine how they affect the ROM of crocodile shoulder joints (Hutson & Hutson, 2013), wrist folding (Hutson & Hutson, 2014) and digits (Hutson & Hutson, 2015). These studies identified that complete soft tissue removal tended to increase the ROM and if separation of the articular surfaces were avoided, underestimates of the potential ROM of dinosaurs and other fossil archosaurs were considered likely (Hutson & Hutson, 2013).

Additionally, there have been non-ROM developments in understanding the extent that cartilage influences limb length and how the joints functioned, although most of these studies focused on sauropod limbs (i.e., Bonnan et al., 2010; Holliday et al., 2010).

The aim of this study is to continually improve on the skeletal reconstruction and palaeobiological knowledge of the Australian theropod *Australovenator wintonensis*. Using the phylogenetic bracket of dinosaurs (EPB) (Witmer, 1995), crocodylians and birds provide an *in vivo* tool to determine the paleobiology and the effect soft tissue has on skeletal ROM. We decided to use *Dromaius novaehollandiae* Latham, 1970 (commonly known as an emu) as our *in vivo* comparison as the pedal morphology of theropods are much closer morphologically than crocodiles. Understanding the soft tissue anatomy and its effects on the skeletal ROM of *Dromaius* pes has enabled a biological reconstruction of the *Australovenator* pes along with estimates of soft tissue ROM, which will be utilised in a future footprint analysis.

The variation in ROM with and without soft tissue in *Dromaius* provided a guide to develop the potential ROM of a fully biologically restored *Australovenator* pes. The dissection of the *Dromaius* pes provided a guide for the replication of the corresponding soft tissue and keratin sheaths of the *Australovenator* pes. Unguals and their corresponding sheaths have been preserved with numerous dinosaur discoveries, some of which include; *Microraptor zhaoianus* Xu, Zhou & Wang, 2000; *Microraptor gui* (Xu et al., 2003); an oviraptorid Clark, Norell & Chiappe, 1999; *Sinornithosaurus millinii* Xu, Wang & Wu, 1999; *Protarchaeopteryx robusta* (Ji et al., 1998), *Caudipteryx zhou* (Ji et al., 1998) and dromaeosaurid NGMC 91 (Ji et al., 2001). These sheaths are preserved as mostly a two-dimensional outline. They provide a visual guide for developing a dorsal and ventral extremity for a sheath. The *Australovenator* sheaths are here reconstructed in 3-D using extant comparisons with *Dromaius novaehollandiae*.

## Material and Methods

### Specimens

The holotype specimens of Australian Age of Dinosaur Fossil (AODF) 604 used in the following analysis include: metatarsals I, II, III & IV; pedal phalanges: I-1 (Fig. S1); I-2 (Fig. S2); II-1 (Fig. S3); II-2 (Fig. S4); II-3 (Fig. S5); III-1 (Fig. S6); III-2 (Fig. S7); III-3 (Fig. S8); III-4 (Fig. S9); IV-1 (Fig. S10); IV-2 (Fig. S11); IV-3 (Fig. S12); IV-4 (Fig. S13); IV-5 (Fig. S14).

The osteological descriptions were adequately described in White et al. (2013a) however their skeletal identifications were not interpreted correctly, therefore we have supplied updated supplementary figures with their correct skeletal identifications and accompanying digital reconstructions. Some of the specimens required restoration of their articular facets due to poor preservation. These include metatarsal IV, pedal phalanges I-1, II-2, IV-1 and IV-2. These specimens were restored digitally by projecting the articular surfaces of the buttressing elements to the damaged surface in Zbrush (see below). The cranial and ventral condyle morphology was manipulated to extend the morphology of the existing bone and where absent references to other well figured allosauroid specimens (Madsen Jr, 1976; Brusatte, Benson & Hutt, 2008) were utilised to replicate the correct morphology.

### Software and scanning

All the holotype pes elements of *Australovenator wintonensis* and the *Dromaius novaehollandiae* pes were CT scanned at Queensland X-ray, Mackay Mater Hospital, Central-Eastern Queensland using a Philips Brilliance CT 64-slice machine producing 0.9 mm slices. Mimics version 10.01 (Materialise HQ, Leuven, Belgium) was used to create three-dimensional meshes of specimens from the CT scans. The *Dromaius* pes was also magnetic resonance imaging (MRI) scanned at Queensland X-ray, Mackay Mater Hospital. Many of the holotype pes elements were damaged during diagenesis and required some restoration which was completed digitally using Zbrush 4R7 (Pixologic Inc, Los Angeles, CA, USA). Zbrush 4R7 was also used as a first step process to determine the ROM which was used in conjunction with Rhinoceros 5.0 (Robert McNeel and Associates, Los Angeles, CA, USA).

#### Dromaius novaehollandiae

The *Dromaius* pes used in this analysis was obtained from an emu farm. Initially, the pes was secured in both flexed and extended positions and CT scanned to determine the ROM with soft tissue.

By adjusting the contrast of the CT scans within Mimics 10.01 it enabled the outer emu skin surface to be displayed which provided a visible external limit for the replication of soft tissue. An MRI scan was also performed with the pes in a plantigrade stance in order to conduct a digital dissection. The resolution was not adequate to decipher the exact ligament and tendon structures so we conducted a physical dissection to ascertain specific soft tissue features more accurately. The de-fleshed bones were then micro CT scanned using a GE Phoenix v[tome]x X-ray micro-computed tomography scanner. The images were reconstructed using datos∖x acquisition version 2.2.1 RTM. X-ray settings for acquisition were 140 kV, 190 µA, 400 ms, sensitivity 2 using a copper filter at 103 µm (optimal resolution to acquire the largest specimen in a single frame). Reconstruction settings were 16 bit at full (setting 1) resolution, with the volume exported to VGStudio Max 2.0.5; VGL version 4.0.0 (37486) for volume slicing and DICOM creation using default settings at maximum resolution. The specimens were rearticulated both manually and digitally to determine ROM without soft tissue. The CT scans revealed the morphology of the *Dromaius* sheaths were similar to the underlying bone, therefore it was assumed to be similar in *Australovenator*. The method of sheath reconstruction is described below.

The soft tissue was replicated using a combination of ‘*ex vivo’* specimen dissection alongside digital ‘*in silico’* restoration (Hammond, Plavcan & Ward, 2016). Initially the MRI scans were converted into 3-D objects files however their actual structure and corresponding attachment points could not be determined due to the resolution of the images. To reconstruct these soft tissues *in silico*, an *ex vivo* dissection was used as a guide. The initial 3-D files created from the MRI scans were imported into Zbrush 4R7. These were used as transparent files so fully restored soft tissue could be created *in silico* to the correct proportions.

#### Australovenator wintonensis

The *Dromaius* dissection was utilized to understand the biomechanics of the *Dromaius* pes in order to help reconstruct the *Australovenator* pes and better estimate its ROM with the addition of soft tissue. The *Dromaius* specimen was scaled to the same proportions as *Australovenator* enabling a more accurate assignment of soft tissue and more comparable ROM. The pedal phalanges of *Australovenator* were digitally articulated along the same orientation as the *Dromaius* pes which represented a plantigrade stance on a flat solid substrate which is here referred to as the neutral position. The scaling of the *Dromaius* pes helped to determine the dimensions of the *Australovenator* soft tissue.

### Ungual and sheath restoration in *Australovenator*

The extent of the *Australovenator* sheaths’ were calculated using the formula *γ*_est_ = 1.54*γ*_*u*_ described in Glen & Bennett (2007). The estimated sheath angle (*γ*_est_) was calculated from the claw angle of the ungual bone (*γ*_*u*_) and multiplying it by 1.54. The claw angle was determined in Rhinoceros 5.0 from the angle created from the distal limit of the flexor tubercle (*B*_*u*_) and the ungual tip (*T*_*u*_).

**Figure 3:**
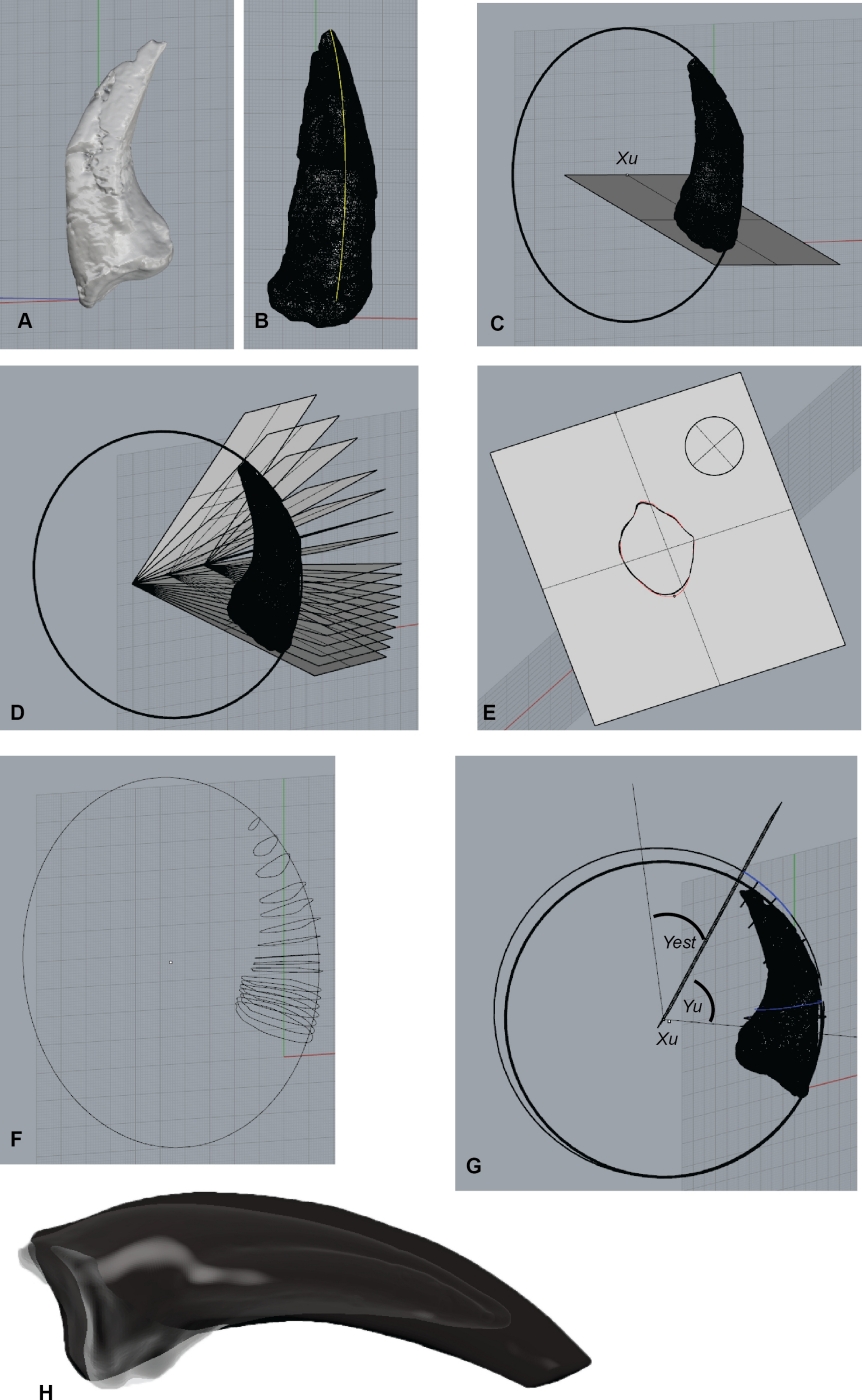
*Australovenator* ungual and sheath restoration. (A) Pedal ungual II-3 imported into Rhinoceros 5.0. (B) Outer surface curve drawn to dorsal ungual surface. (C) Circle attached to the best fit of outer curve of the ungual. (D) Section planes drawn from a centre point of the drawn circle. (E) Section through the ungual displaying the outer surface of the ungual. (F) Cross-section ribs of the ungual redrawn to represent unbroken sections. (G) A perfect circle was dorsally offset to the claw’s rim for sheath thickness in Rhinoceros 5.0. The offset synthesized the dorsal sheath thickness which was progressive until the sheath’s tip. (H) Wear facets on te reconstructed sheaths. Abbreviations: Centre of circle (*Xu*), Ungual angle (*Yu*), Sheath extent (*Yest*).

The dorsal guide for the sheath was developed using a circle, which was identified as being accurately fitted to the dorsal rim of birds’ claws (Pike & Maitland, 2004; Glen & Bennett, 2007). A perfect circle was dorsally offset to the claw’s rim for sheath thickness in Rhinoceros 5.0. The offset synthesized the dorsal sheath thickness which was progressive until the sheaths tip. Multiple cross-sections of the ungual and the dorsal off-set were created using cutting plains developed within Rhinoceros 5.0. These cutting plains were projected from the centre of the ungual circle and were used to section the ungual at multiple intervals. The cross-sections were used as a guide to draw the outer sheath cross-section outlines. The morphology of the *Dromaius* sheaths were similar to the underlying bone therefore the assumption is here made that they were also similar in *Australovenator*. These created an outer sheath rib cage which along with the dorsal off-set guide and the underlying ungual were exported into Zbrush 4R7 to replicate the sheath. The restored sheaths are replicated to a distinct point however wear is quite evident on the *Dromaius* sheaths. The wear facets were replicated on the *Australovenator* sheaths from removing the distal portions of the sheaths along an artificial sub straight surface plain that the articulated foot was created on in Zbrush (Fig. 3).

### Range of motion measurements

**Figure 4:**
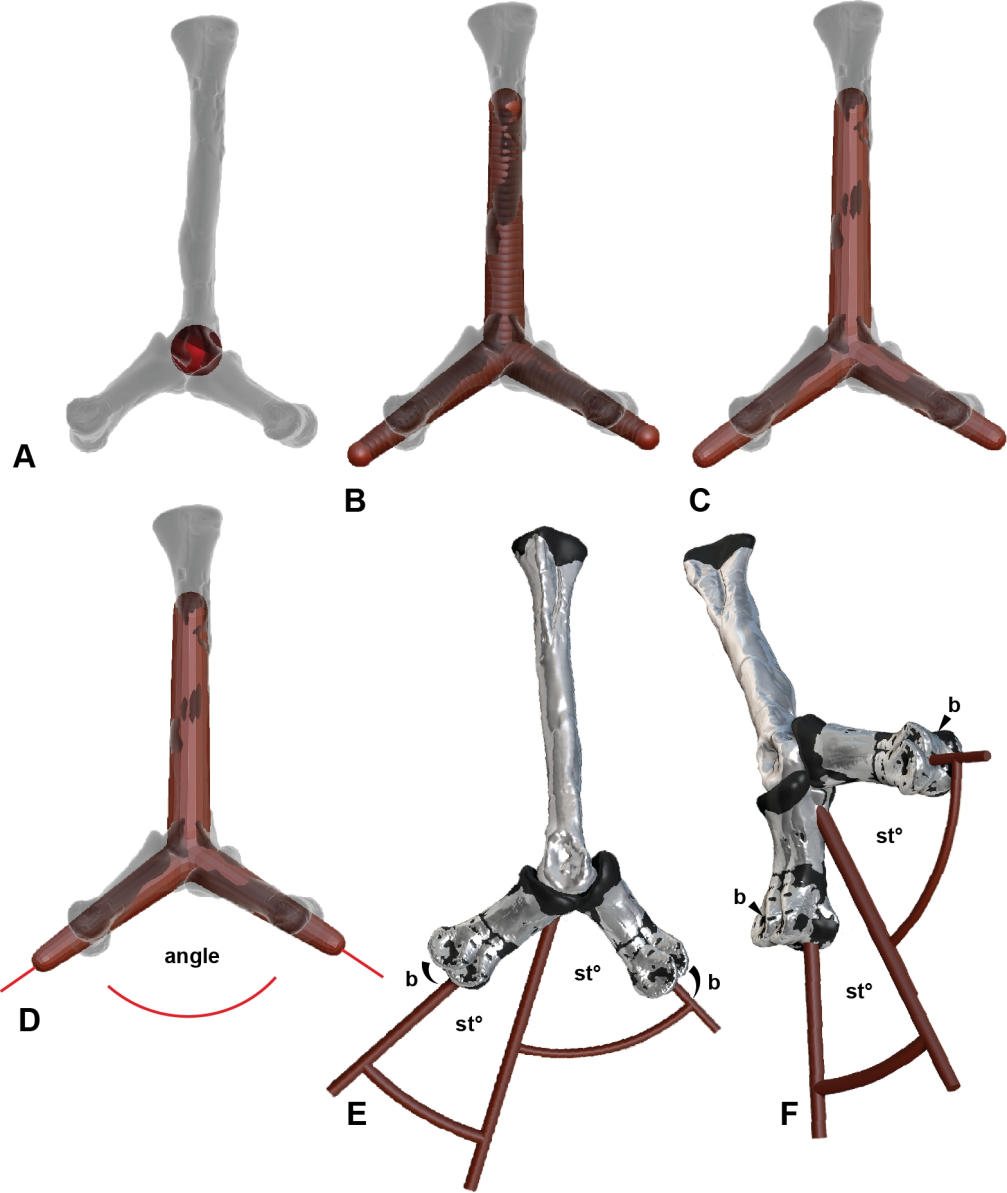
Determining the ROM using Zbrush and Rhino 5.0 using metatarsal II (MTII) and II-1 as an example. (A) Metatarsal II and phalanx II-1 in extended and flexed positions with a sphere representing the range of motion surface of the condyle end of MTII. (B) Separate overlapping spheres drawn as attachments to the central sphere which bisecting along the axis of the distal positions of II-1. (C) Converted from sphere to a mesh. (D) The mesh exported from Zbrush 4R7 into Rhinoceros 5.0. Lines are drawn centrally dissecting the meshes so the ROM can be determined using the angle function. (E) Medial view of a soft tissue articulation angle. The soft tissue angle (Table 2) is replicated with a 3-D protractor with the joint’s corresponding soft tissue angle, which was aligned with the interphalangeal rotation point. The phalanx is re-oriented to its suspected soft tissue articulation. (F) Oblique view of soft tissue ROM adjustment. Abbreviations: bone (b) ROM; st^o^, soft tissue ROM angle.

The ROM limitations of both *Australovenator* and *Dromaius* were determined with a combination of ‘*ex vivo*’ manual specimen articulation alongside digital ‘*in silico*’ articulation (Hammond, Plavcan & Ward, 2016) using both Rhinoceros 5.0 and Zbrush 4R7 (Fig. 4). The maximum soft tissue extension and flexion limits were obtained by bending the digits to their maximum ROM without breakage and securing these positions for CT scanning.

There remains some potential ROM error since the foot of the *Dromaius* specimen was severed from the rest of the leg, meaning tension in a number of tendons was relaxed, and subsequently increased potential ROM. This highlights the impact soft tissue has on ROM in the pes. The severed limbs were unavoidable for this study due to the complications involved in scanning a complete *Dromaius*, however scans are planned for the future which will focus on the entire limb ROM.

The soft tissue ROM was simulated by CT scanning a fleshed *Dromaius* pes poised with the metatarsus in a vertical position simulating a walking stance on a flat substrate. The ROM was determined from this neutral position (0°). This reduced the extension values and increased the flexion capabilities however the overall ROM remained the same. The neutral position was simulated with the *Australovenator* specimens by superimposing the reconstructed pes over the digitally scaled up *Dromaius* pes (Table 1).

**Table 1:** Therpod peda ROM. ROM value of extension (top numbers) and flexion (bootom numbers) of theropod pedal phalanges not allowing for soft tissue.

Dinosaur taxa	ROM	I-1	1-2	II-1	II-2	II-3	III-1	III-2	III-3	III-4	IV-1	IV-2	IV-3	IV-4	IV-5	Information source
*Australovenator*	Extension	49^a^	23^a^	58	41	32^a^	65	64^a^	30	44	62^a^	43	42^a^	49	51	AODF604
	Flexion	49^a^	23^a^	52	42	26^a^	68	53^a^	59	42	84^a^	31	51^a^	58	35	
	ROM	**98**	**46**	**110**	**83**	**58**	**133**	**117**	**89**	**86**	**146**	**74**	**93**	**107**	**86**	
*Dilophosaurus*	Extension	40	–	38	50	10	50	36	43	30	23	47	34	35	18	Senter (2009)
	Flexion	9	–	37	40	55	50	72	79	62	42	30	65	39	55	
	ROM	**49**	**0**	**75**	**90**	**65**	**100**	**108**	**122**	**92**	**65**	**77**	**99**	**74**	**73**	
*Allosaurus*	Extension	62	0	27	100	14	74	82	53	28	33	65	48	53	53	Senter (2009)
	Flexion	15	40	38	66	54	45	66	83	34	27	62	65	60	−4	
	ROM	**77**	**40**	**65**	**166**	**68**	**119**	**148**	**136**	**62**	**60**	**127**	**113**	**113**	**49**	
*Mononykus*	Extension	30	25	59	77	73	57	79	54	73	58	65	58	43	83	Senter (2009)
	Flexion	58	53	69	69	62	57	81	67	48	88	34	63	38	64	
	ROM	**88**	**78**	**128**	**146**	**135**	**114**	**160**	**121**	**121**	**146**	**99**	**121**	**81**	**147**	
*Chirostenotes*	Extension	–	2	46	60	13	49	30	22	4	44	76	73	72	−14	Senter (2009)
	Flexion	–	42	41	55	57	59	54	60	45	43	33	51	50	77	
	ROM	**0**	**44**	**87**	**115**	**70**	**108**	**84**	**82**	**49**	**87**	**109**	**124**	**122**	**63**	
*Deinonychus*	Extension	–	10	59	90	40	65	62	58	33	56	30	58	51	9	Senter (2009)
	Flexion	–	74	34	14	67	54	68	57	70	44	77	60	78	37		
	ROM	–	**84**	**93**	**104**	**107**	**119**	**130**	**115**	**103**	**100**	**107**	**118**	**129**	**46**	
*Bambiraptor*	Extension	–	0	66	128	−15	76	75	78	10	61	60	65	66	7	Senter (2009)
	Flexion	–	90	73	36	80	95	105	71	87	74	88	89	102	75	
	ROM	–	**90**	**139**	**164**	**65**	**171**	**180**	**149**	**97**	**135**	**148**	**154**	**168**	**82**	

**Notes.**

a Estimated ROM value due to poor preservation.

**Table 2:** ROM values of *Dromaius* pes with and without soft tissue. The variation in ROM value (Variance) is applied to the ROM value of the *Australovenator* specimens to simulate extension and flexion allowing for soft tissue.

Specimens	ROM	II-1	II-2	II-3	III-1	III-2	III-3	III-4	IV-1	IV-2	IV-3	IV-4	IV-5
*Dromaius* ST	Ext	41	2	3	18	18	27	2	25	8	19	2	2
	Flex	118	73	42	114	64	35	17	114	41	16	19	14
*Dromaius* bone	Ext	49	15	14	27	46	40	22	68	21	22	14	16
	Flex	126	77	50	122	107	93	55	119	91	48	39	28
*Dromaius* variance		8	13	11	9	28	13	20	41	13	3	12	14
		8	4	8	8	43	58	38	5	50	32	20	14
Percentage		0.16	0.87	0.79	0.33	0.61	0.33	0.91	0.62	0.62	0.14	0.86	0.88
		0.06	0.05	0.16	0.07	0.40	0.62	0.69	0.04	0.55	0.67	0.51	0.50
*Australovenator*	Ext	37	4	27^a^	20	66^a^	67	39	47^a^	3	39^a^	50	55
	Flex	63	92	41^a^	108	57^a^	58	36	94^a^	68	60^a^	57	31
*Australovenator* variance		6	3	21	7	40	22	35	29	2	5	43	48
		4	5	7	7	23	36	25	4	37	40	29	16
*Australovenator* ST	Ext	31	1	6	13	26	45	4	18	1	34	7	7
	Flex	59	87	34	101	34	22	11	90	31	20	28	16

**Notes.**

Abbreviations Ext Extension Flex Flexion ST Soft tissue

a Estimated ROM value due to poor preservation.

To arrange the pedal elements to allow for soft tissue extension and flexion in *Australovenator* a 3-D protractor was created in Rhinoceros 5.0. This was done by using the ‘Arc’ tool to create the specific angle required. Poly lines were drawn to intersect the arc or circles centre and then all of the lines were selected to convert to a ‘pipe’ or 3-D mesh in the form of the required 3-D protractor angle. These protractor angles were imported into Zbrush 4R7, which represented the calculated soft tissue extension and flexion values of the *Australovenator* specimens from Table 2.

### Ethics statement

All necessary permits were obtained for the described study, which complied with all relevant regulations. Permission to excavate the specimens from Elderslie station was obtained from the landholders who donated all specimens to the Australian Age of Dinosaur Museum of Natural History (AAOD). During excavation, each specimen was given a preliminary field number for location and storage purposes. All specimens pertaining to the holotype *Australovenator wintonensis* are allocated the specimen number AODF604. The specimens are stored in climate controlled conditions at the Australian Age of Dinosaurs Museum 15km east of Winton, Queensland, Australia. The *Dromaius* pes, which was an offcut from farming emu oil, was purchased from an emu farm for the sole purpose of this analysis. No permits were required as this animal is not endangered and was farmed.

## Results

### Revised skeletal identification of the *Australovenator* pes

A review of prior publications (Hocknull et al., 2009; White et al., 2012; White et al., 2013a) revealed a number of discrepancies in the initial phalanx identification of *Australovenator*.

These include: a small phalanx that was initially described as manual phalanx II-2 (see Figs. 28J–28O in Hocknull et al., 2009) and later reallocated to the position of manual phalanx III-1 (see Figs. 2, 21 in White et al., 2012) is here repositioned to its correct position as a pedal phalanx III-3; a second pedal phalanx described as pedal phalanx III-2 (see Figs. 16, 24 in White et al., 2013a) is repositioned as a left pedal phalanx III-1; the pedal phalanges (left and right) (see Fig. 17 in White et al., 2013a) that were replaced with the newly assigned pedal phalanx III-3 are repositioned as pedal phalanges III-2. The initial misidentification of III-I was based on the poor preservation of the specimen in particular the distal end (see Fig. 15 in White et al., 2013a), which appeared wider than the specimen that was initially identified as pedal phalanx III-2. Two of the unguals were also repositioned. Originally identified as pedal phalanx III-4 (see Fig. 18 in White et al., 2013a), it is now correctly interchanged with pedal phalanx II-3 (see Fig. 14 in White et al., 2013a). A new pedal phalanx recently prepared belongs to pedal phalanx I-1 (Fig. S1). It is poorly preserved and required extensive reconstruction. The ventral half of the proximal articular surface is preserved; however, neither medial nor lateral facets could be distinguished. The main shaft is compressed and shattered. The medial condyle is better preserved than the lateral and possesses a deep fossa. It also possesses portions of its distal surface enabling the entire length to be determined. Based on the remnants of the distal surface it appears that the medial condyle is slightly taller than the lateral condyle however it’s indeterminate which condyle was broader (Measurements in Table S1).

### ROM analysis

The ROM of each interphalangeal joint articulates in mostly the plane of extension and flexion however the metatarsophalangeal joint allows for adduction and abduction. The digits of *Australovenator* converge during flexion and spread apart during hyper-extension which is consistent with most theropods apart from deinonychosaurs (Senter, 2009). The abduction of metatarsal IV is questionable due to the poor preservation of its distal end. The three-dimensional digital articulation of the other elements not only assisted in the specimen’s reconstruction but helped more accurately estimate its ROM capabilities. Some of the *Australovenator* specimens have poorly preserved articular facets. Without reconstruction their ROM could not have been accurately determined. In these cases the ROM still remains a best estimate only.

**Figure 5:**
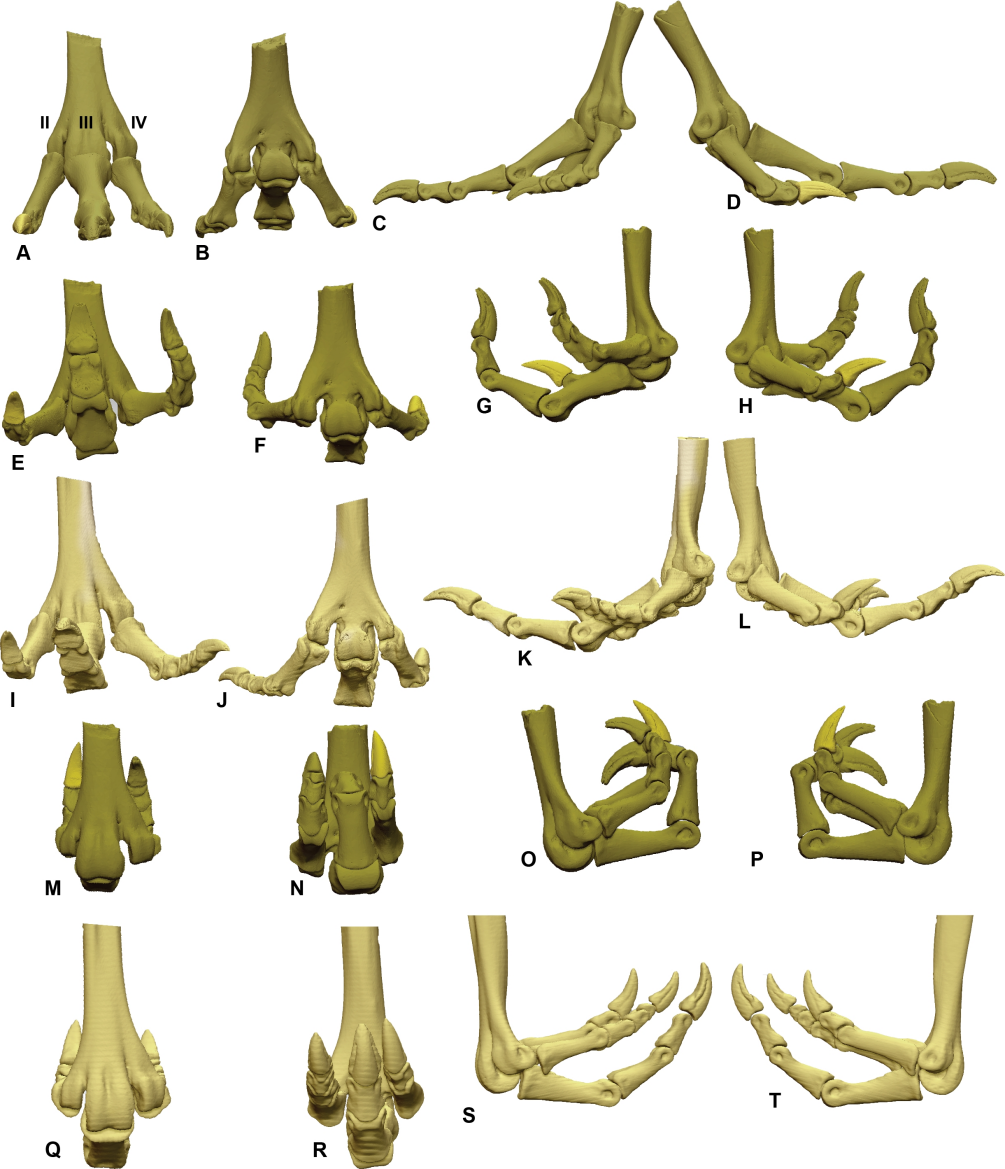
ROM of the *Dromaius* pes with and without soft tissue. Neutral posture: (A) Anterior; (B) Plantar; (C) Lateral; (D) Medial. Extended without soft tissue: (E) Anterior; (F) Plantar; (G) Lateral; (H) Medial. Flexed without soft tissue: (I) Anterior; (J) Plantar; (K) Lateral; (L) Medial. Extended with soft tissue: (M) Anterior; (N) Plantar; (O) Lateral; (P) Medial. Flexed with soft tissue: (Q) Anterior; (R) Plantar; (S) Lateral; (T) Medial.

**Figure 6:**
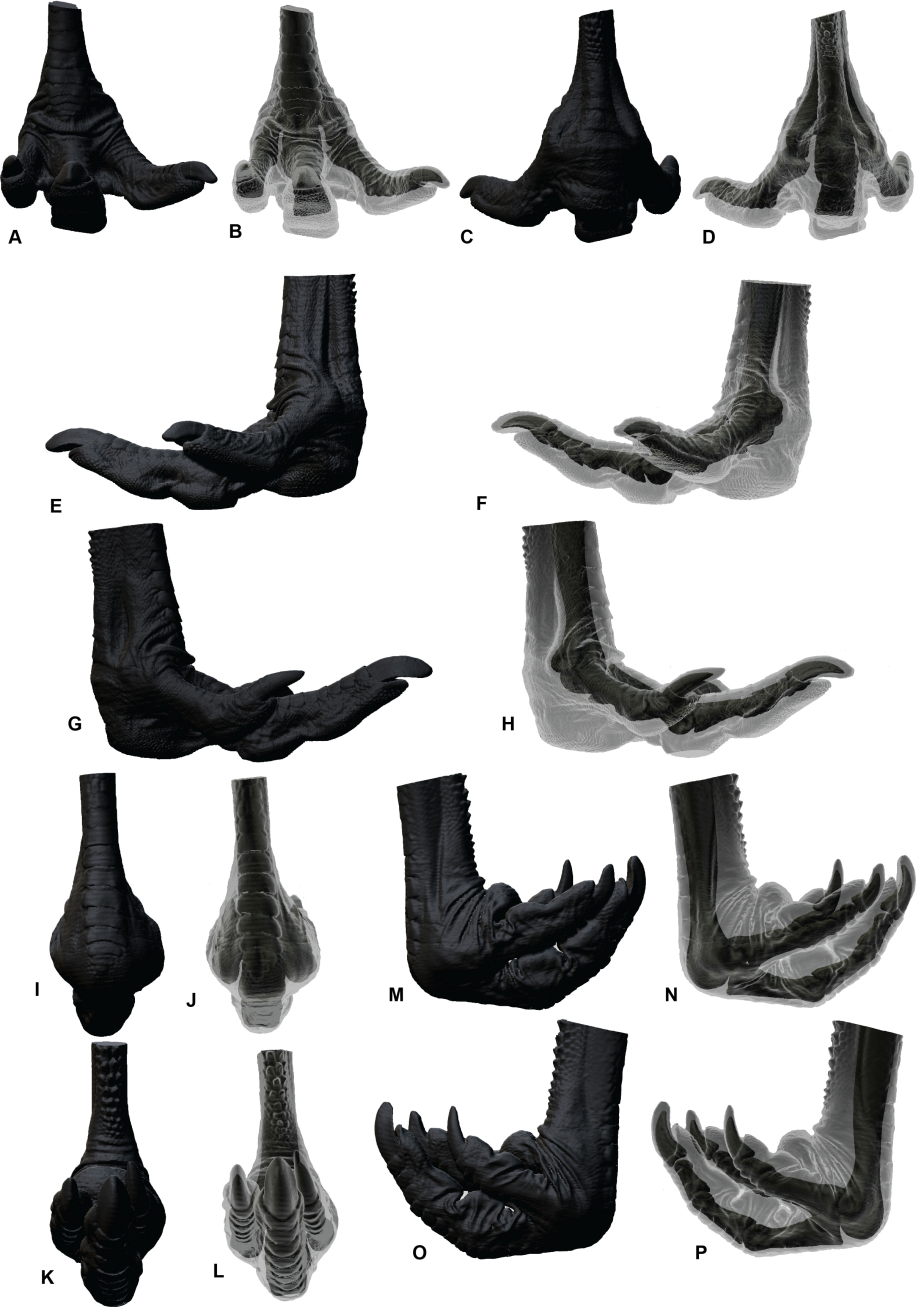
Soft tissue ROM of the * Dromaius* pes. Extended in: (A, B) Cranial; (C, D) Plantar; (E, F) Medial; (G, H) Lateral. Flexed in: (I, J) Cranial; (K, L) Plantar; (M, N) Medial; (O, P) Lateral.

The ROM of the *Dromaius* pes revealed distinct variation with and without the presence of soft tissue. The lowest ROM variation was the flexion of the first phalanx of each digit (II-1, III-1 and IV-1) (Table 2). The largest ROM variation comprised the unguals where the bone ROM far exceeded the soft tissue ROM. There was a small amount of ROM variation between II-1 and II-2 in both flexion and extension. Distinct variation exists in the extension capabilities of II-1, III-2, III-3, IV-1, IV-2, IV-3 and IV-4 with the presence of soft tissue (Table 2). The variation in ROM capabilities with the addition of soft tissue was used to allocate soft tissue ROM to the *Australovenator* pes (Figs. 2, 5, 6 and Table 2).

### Dissection of the *Dromaius* pes and reconstruction of the *Australovenator* pes

Herein the soft tissue of *Dromaius* and hypothetical soft tissue features of the *Australoventor* pes is described along with their effect on the pedal ROM.

The *Dromaius* phalanges and the metatarsals possess cartilaginous caps on both their proximal and distal ends. Additionally the ROM of the *Dromaius* pedal elements were found to be more extensive without cartilage. This is because the proximo-ventral cartilaginous extensions of the phalanges to limit flexion and extension. Interestingly similar findings were also demonstrated in the finger bones of Alligators (see Fig. 2H in Hutson & Hutson, 2015).

**Figure 7:**
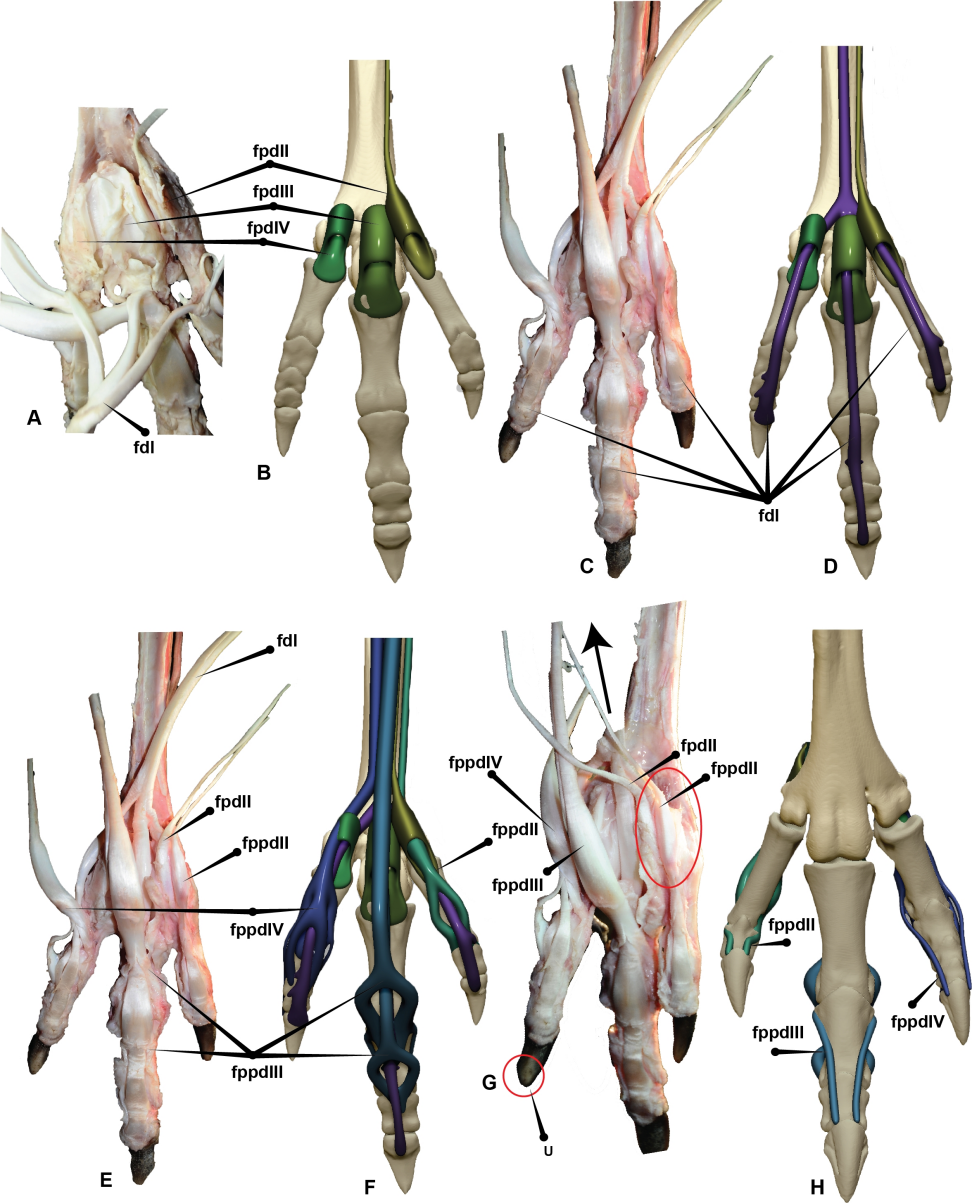
*Dromaius* pes with intermediate, superficial and deep flexors. Plantar view displaying: (A) Extension of superficial flexors for each corresponding digit that housed the deep and intermediate flexors. (B) Corresponding digital render of superficial flexors. (C) Ventral dissection identifying the superficial flexors (cartilage housing for the tendons) and the * flexor digitorum longus*. (D) Corresponding digital rendering of dissection. (E) Ventral dissection identifying intermediate, superficial and deep tendons. (F) Corresponding digital rendering of dissection. (G) Close up of the digit II’s superficial flexor. (H) Cranial view of the intermediate flexor attachments. Abbreviations: *M. flexor digitorum longus* (fdl); *M. flexor hallucis brevis* (fhb); *M. flexor hallucis longus* (fhl); *M. extensor digitorum longus* (edl); *M. flexor perforatus digiti II* (fpdII); *M. flexor perforans et perforatus digiti II* (fppdll); *flexor perforates digiti III* (fpdIII); *M. flexor perforans et perforatus digiti III* (fppdIII); *flexor perforatus digiti IV* (fpdIV).

**Figure 8:**
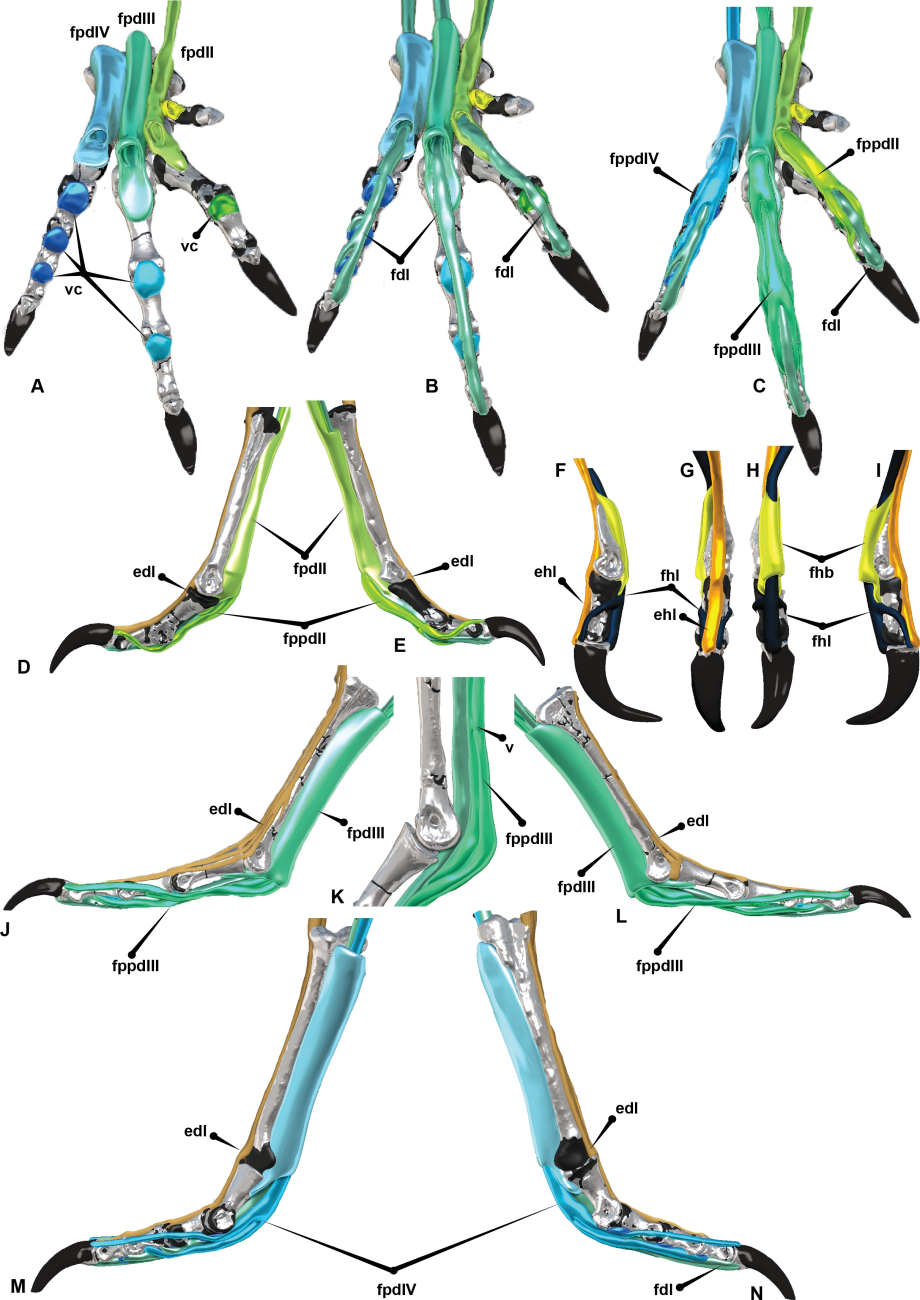
*Australovenator* pedal digits I–IV with reconstructed intermediate, superficial and deep flexors. Plantar view displaying: (A) Extension of proximo-ventral cartilage of each phalanx and superficial flexors for each corresponding digit (vc). (B) *Deep flexor digitorum longus*. (C) Superficial flexors, intermediate flexors and deep flexors combined. Digit II with *flexor digitorum longus*, *flexor perforatus digiti II* and *flexor perforans et perforatus digiti II*, (D) Lateral, (E) Medial. Digit I with *flexor hallucis brevis*, *flexor hallucis* and *extensor digitorum longus*, (F) Medial, (G) Lateral, (H) Plantar, (I) Cranial. Digit III with *flexor digitorum longus*, *flexor perforatus digiti III*, *flexor perforans et perforatus digiti III*; *Flexor perforatus digiti III* and *flexor perforans et perforatus digiti III* connected by the *fibrous vinculum:* (J, K) Lateral, (L) Medial. Digit IV with *flexor digitorum longus* and *flexor perforatus digiti IV*: (M) Lateral, (N) Medial. Abbreviations: *M. flexor digitorum longus* (fdl); *M. flexor hallucis brevis* (fhb); *M. flexor hallucis longus* (fhl); *M. extensor digitorum longus* (edl); *M. flexor perforatus digiti II* (fpdII); *M. flexor perforans et perforatus digiti II* (fppdll); *M. flexor perforatus digiti III* (fpdIII); *M. flexor perforans et perforatus digiti III* (fppdIII); *M. flexor perforatus digiti IV* (fpdIV).

The digital flexor muscles consist of intermediate flexors (*M. flexor perforans et perforatus digiti II* (FPPDII) and *M. flexor perforans et perforatus digiti III* (FPPDIII)); superficial flexors (*M. flexor perforatus digiti* II (FPDII), *M. flexor perforatus digiti III* (FPDIII) and *M. flexor perforatus digiti IV* (FPD IV)) and deep flexors (*M. flexor hallucis longus* (FHL) and *M. flexor digitorum longus*(FDL)) (Getty, 1975; Lamas, Main & Hutchinson, 2014; Patak & Baldwin, 1998) (Figs. 7 and 8). The superficial flexor tendons FPDII and FPDIII insert proximally on the respective digit and are perforated by the corresponding intermediate flexor tendon, whereas the fourth is perforated by only the FDL. The intermediate flexor tendons FPPDII and FPPDIII are perforated by the other respective branches of the FDL. Interestingly the *Dromaius* dissection revealed that the manual movement of FPDII dictated movement of the digit. Unlike FPDIII and FPDIV, the superficial flexors FPDII was not distally attached to the plantar surface (Fig. 7).

The long digital flexor tendon of the hallux perforates the tendon of a short flexor muscle in the tarsometatarsus. FPDII inserts on the ventral surface of the metatarsophalangeal joint capsule of the second digit. The FPDII houses the FPPDII and the FDL. The latter perforates the superficial flexor resulting in medial and lateral tendons which wrap around from the basal attachment. It anchors cranially posterior to the condyle head of pedal phalanx II-1 and terminates with a cranio-proximal attachment to the ungual on either side of the *extensor digitorum longus* (EDL). The superficial flexor unifies the digit into a functional unit preventing hyperextension. FPDIII inserts on the metatarsophalangeal joint capsule of the third digit. Medial and lateral extensions of the FPDIII wrap from the ventral attachment proximal to the distal condyles of pedal phalanx III-2 and migrate distally to a cranial attachment on the proximal end of pedal ungual III-4 on either side of the EDL. This prevented over hyperextension of the digit. FPDIII is perforated by the FPPDIII which attach to the proximal portion of pedal phalanx III-3 and the first interphalangeal joint. These two tendons are connected by a *fibrous vinculum* (FV). The intermediate flexor is perforated by the FDL. It inserts behind the condyles of the phalanges, at the third interphalangeal joint and the proximal portion of the ungual. FPDIV attaches to the distal base of the first phalanx and subdivides into four separate tendons, anchoring on the first, second, third and fourth interphalangeal joints. The first attaches ventrally on the metatarsophalangeal joint. The second inserts on the first interphalangeal joint and attaches latero-ventrally to pedal phalanx IV-3. The third and fourth tendons anchor at each interphalangeal joint and wrap the digit from the ventral to cranial surface attaching to either side of the EDL on the cranial surface of the ungual. FPDIV is perforated by the FDL.

The FPDIV inserts on the plantar surface, proximal of the distal condyles of pedal phalanges IV-2, IV-3, IV- 4, the third and fourth interphalangeal joints and attaches to the proximo-ventral side of the ungual (Figs. 7 and 8).

**Figure 9:**
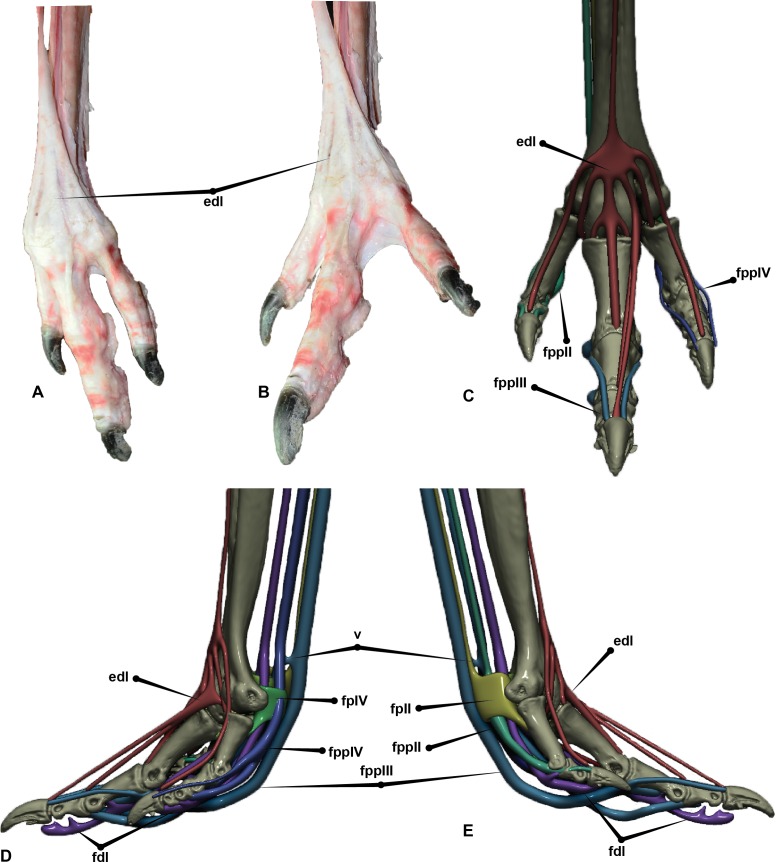
Extensors of the *Dromaius* pes. (A) *Dromaius* pes in suspended flexed position; (B) *Dromaius* pes in extended position; (C) Digital render of *extensor digitorum longus* in cranial view. *Dromaius* pes with dissection in exploded view; (D) Lateral (E) Medial. Abbreviations: *M. flexor digitorum longus* (fdl); *M. flexor hallus brevis* (fhb); *M. flexor hallucis longus* (fhl); *M. extensor digitorum longus* (edl); *M. flexor perforans II* (fpdII); *M. flexores perforans et perforates digiti II* (fppdll); *M. flexor perforans III* (fpdIII); *M. flexores perforans et perforates digiti III* (fppdIII); *M. flexor perforans IV* (fpdIV).

**Figure 10:**
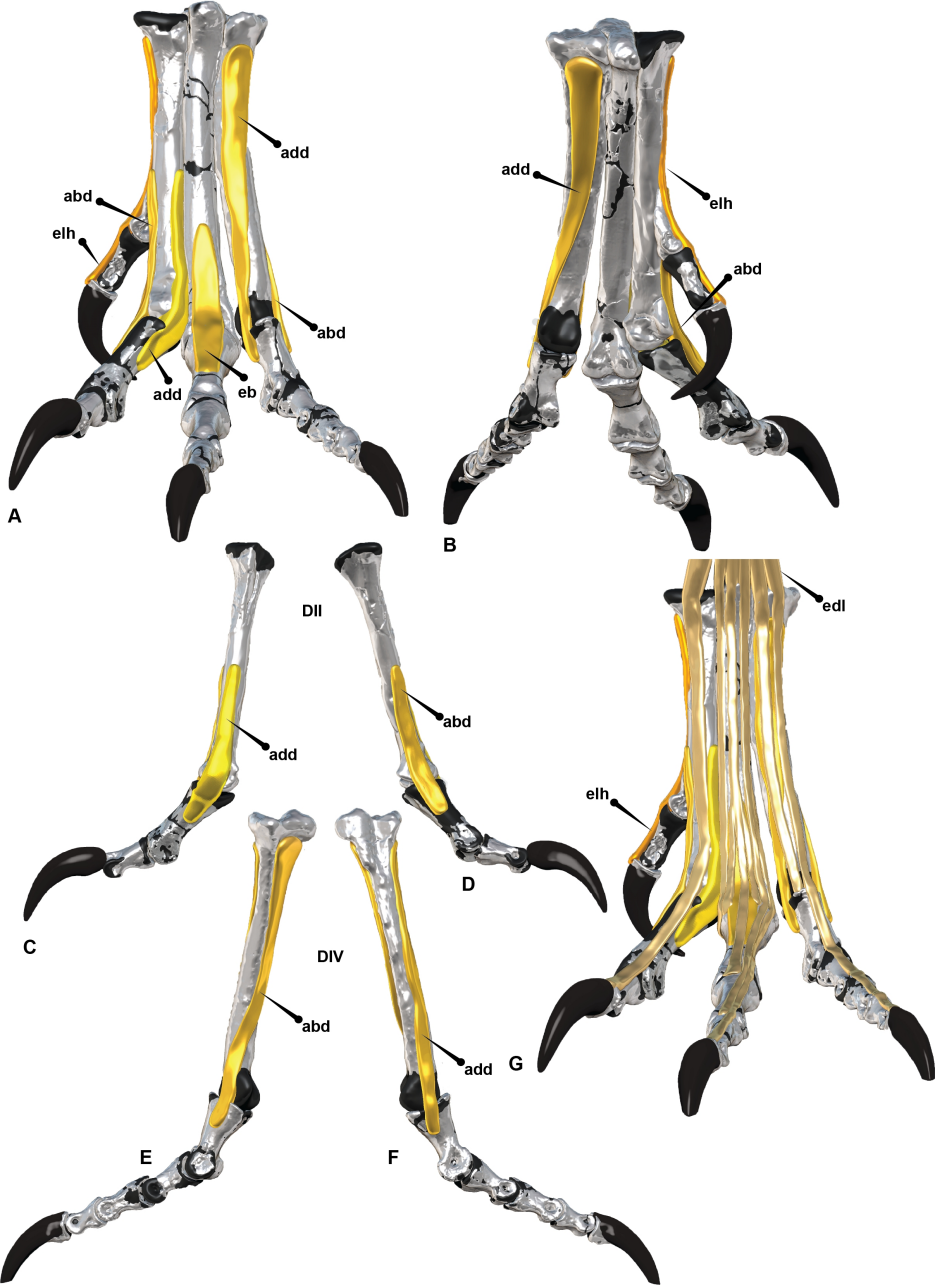
Adductor and abductor tendons of the *Australovenator* pes. (A) Cranial; (B) Plantar; (C) *Adductor digiti II*; (D) *Abductor digiti II*; (E) *Abductor digiti IV*; (F) *Adductor digiti IV*; (G) Extensors: *extensor digitorum longus* (edl), *extensor hallucis longus* (elh). Abbreviations: *adductor* (add), *abductor* (abd), *extensor brevis digiti III* (eb).

The EDL trifurcates from a small canal which runs along the cranial surface of the tarsometatarsus supplying single or double tendons to the second, third and fourth digits (Fig. 9). This same feature is hypothesised to have trifurcated from the ascending process of the astragalus in *Australovenator* (Fig. 10G). It functions as the extensor of the second, third and fourth digits (Getty, 1975; Lamas, Main & Hutchinson, 2014; Patak & Baldwin, 1998). Small fibres of the EDL insert on the cranio-distal ends of the pedal phalanges and the cranio-proximal ends of phalanges and unguals. When the tendon is pulled all of the digits extend simultaneously (Fig. 9).

Digit I is absent in *Dromaius* therefore a direct muscle comparison with *Australovenator* was not possible. Therefore *Dendragapus obscurus* Brooks, 1929 (blue grouse) was used as a biological comparison from Getty (1975). The *extensor hallucis longus* (EHL) attaches to the cranial surface of the second metatarsal and migrates to the cranial aspects of the first digit, anchoring at the metatarsophalangeal joint of metatarsal 1 and attaching to the proximal end of the terminal ungual. Its function is to extend the first digit. The *flexor hallucis brevis* (FHB) originates from the medial aspect of metatarsal 1 and attaches to the proximal plantar end of pedal phalanx I-1. It’s perforated by the FHL. A medial and lateral arm of the FHL is suspected to have wrapped from the plantar attachment and migrated dorsally behind the distal condyles of pedal phalanx I-1.

The anchor points were at the inter-ungual joint and attach to the proximo-cranial end of the ungual on either side of the EHL (Getty, 1975; Lamas, Main & Hutchinson, 2014; Patak & Baldwin, 1998) (Figs. 8F–8I).

Abductor digiti II originates on the distal half of the medial aspect of metatarsal II and attaches to the medial surface of pedal phalanx II-1. The adductor digiti II originates on the distal lateral surface of metatarsal II forcing an adduction of digit II. *Extensor brevis digiti III* (EBDIII) originates on the dorsal surface of metatarsal III and attaches to the cranio-proximal end of pedal phalanx III-1. It assists in the extension of digit III. *Extensor brevis digiti IV* (EBDIV) originates from the dorsolateral surface of metatarsal IV and migrates between metatarsals III and IV attaching on the proximo-medial side of the pedal phalanx IV-1. It assists in the fourth digit’s extension. Abductor digiti IV originates on the lateral surface of metatarsal IV near the proximal end and migrates along the lateral surface to attach to the lateral surface of pedal phalanx IV-1. It assists in the abduction of the fourth digit and the extension of pedal phalanx IV-1 (Figs. 9 and 10).

**Figure 11:**
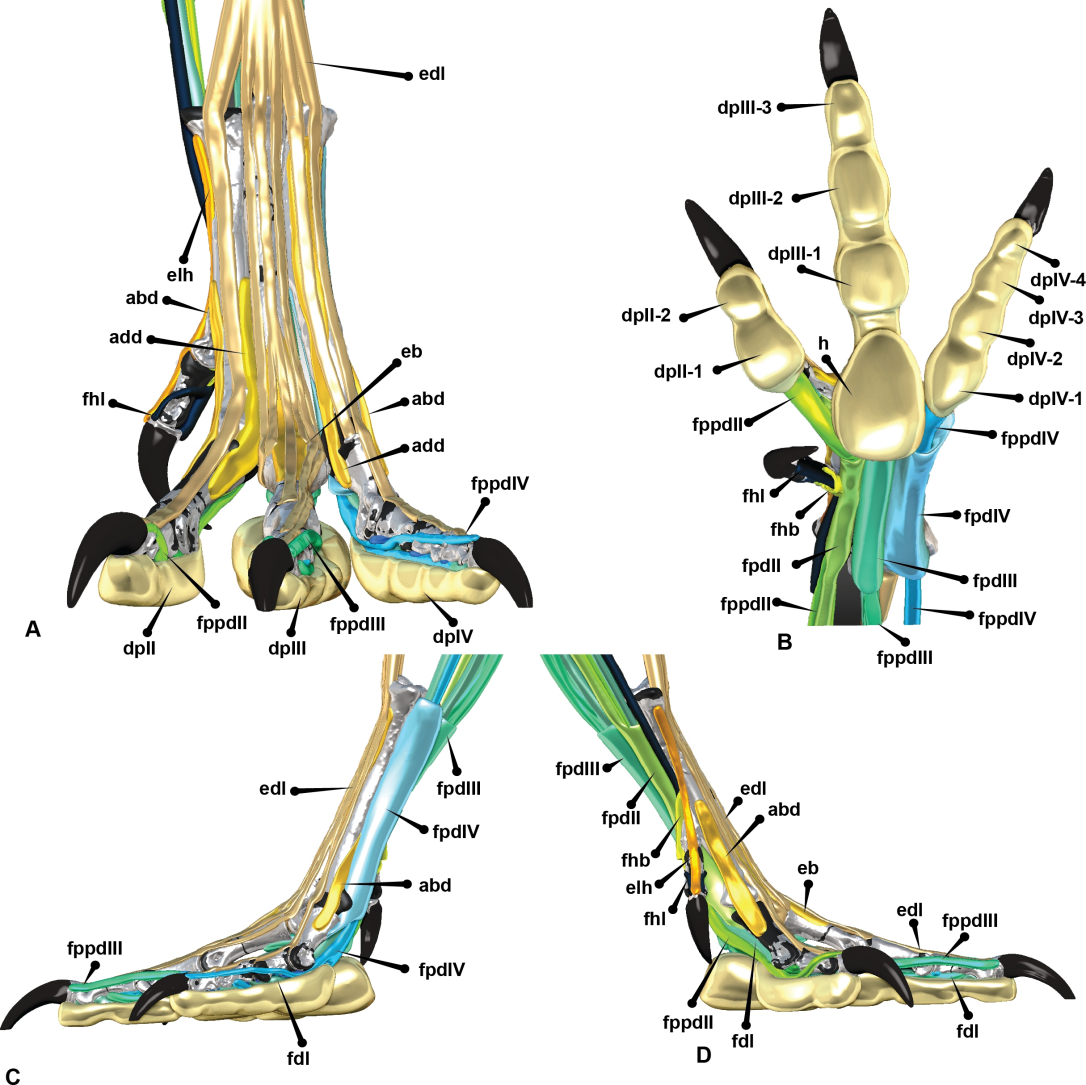
Reconstructed pedal biology. (A) Cranial. (B) Plantar. (C) Lateral. (D) Medial. Abbreviations: *M. flexor digitorum longus* (fdl); *M. flexor hallucis brevis* (fhb); *M. flexor hallucis longus* (fhl); *M. extensor digitorum longus* (edl); *M. flexor perforatus digiti II* (fpdII); *M. flexor perforans et perforatus digiti II* (fppdll); *M. flexor perforatus digiti III* (fpdIII); *M. flexores perforans et perforatus digiti III* (fppdIII); *M. flexor perforatus digiti IV* (fpdIV), *vinculum* (v).

In *Dromaius* the plantar pads are broadest beneath the interphalangeal joints and are thickest towards the proximal part of the foot. Ichnological evidence has indicated that digit IV in theropods possessed four pads (El-Gendy, Derbalah & ElMagd, 2012; Pu et al., 2013; Thulborn, 1990). Additionally, seven large cranial scales exist on pedal digit II and IV, sixteen exist on the third digit and larger cranial scales cover the metatarsus. On the plantar side of the *Dromaius* metatarsus the scales gradually become larger and more pronounced proximally. The ungual sheaths also have distinct wear facets created from constant contact with the substrate, which is more pronounced on digits III and IV with digit II remaining slightly sharper (Figs. 6 and 11).

Similar podotheca morphological features have also been discovered in the fossilised pes of *Concavenator corcovatus* (Ortega, Escaso & Sanz, 2010) from the Lower Cretaceous of Cuena, Spain (Cuesta et al., 2015). They share a common ancestor along the Carcharodontosaurian lineage as per a resent phylogeny provided in Cuesta et al. (2015).

This common ancestor is quite basal to these groups however Cuesta et al. (2015) described the actual morphology of the skin impressions as resembling extant cursorial birds which justifies our use of *Dromaius* and *Concavenator* to reconstruct the skin.

**Figure 12:**
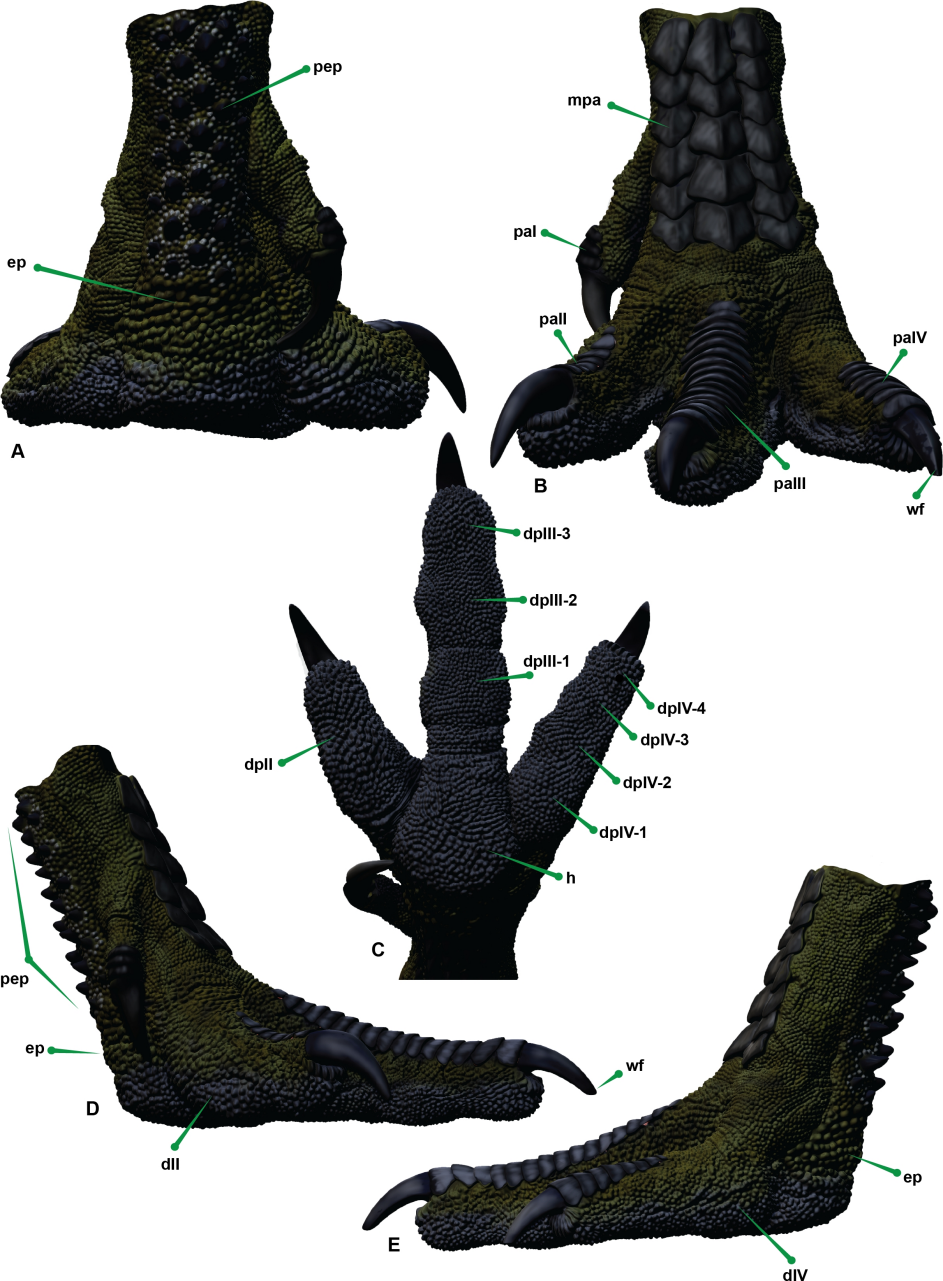
Reconstructed left pes of *Australovenator*. (A) Proximal. (B) Cranial. (C) Plantar. (D) Medial. (E) Lateral view. Abbreviations: enlarged papillae ep; digit I DI; digit II DII; digit III DIII; digit IV DIV; digit pad (dp) (II, III-1, III-2, III-3, IV-1; IV-2, IV-3, IV-4); Digit podotheca (pa); main heal pad (h); metatarsus podotheca (mpa); proximal enlarged podotheca (pep); ungual wear facet (wf).

**Figure 13:**
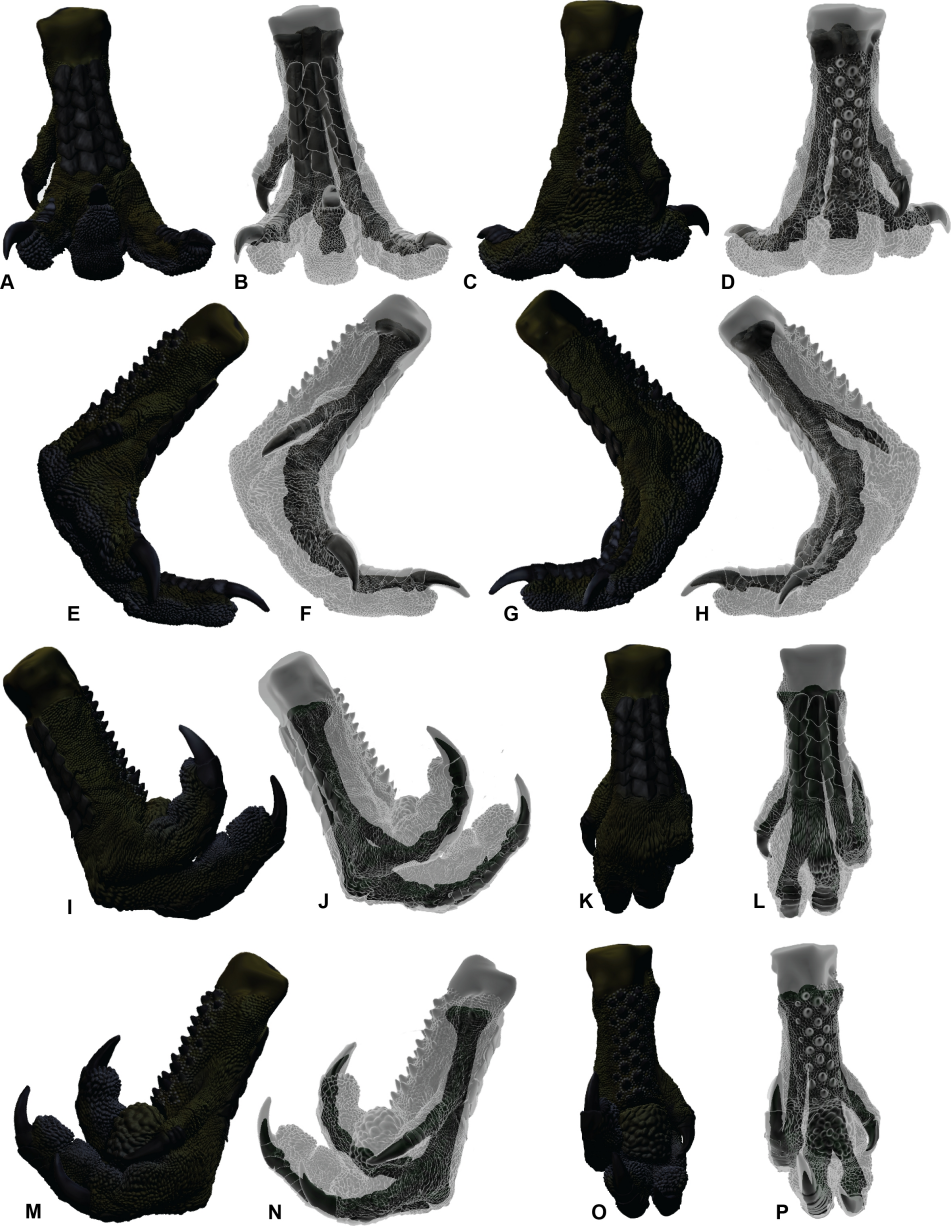
Soft tissue ROM of the *Australovenator* pes. Extended in: (A, B) Cranial; (C, D) Plantar; (E, F) Medial; (G, H) Lateral. Flexed in: (I, J) Lateral; (K, L) Cranial; (M, N) Medial; (O, P) Plantar.

*Concavenator* has papillae impressions that are closely spaced and are less than 1 mm wide. They have a slightly negative epirelief and a linear pattern perpendicularly orientated to the long axis of the phalanx (Cuesta et al., 2015). The smallest scales were irregular in shape and predominately on the ventral surface whereas the medium sized scales progress in size on the lateral side of digit IV. The plantar pads revealed the presence of small papillae which Cuesta et al. (2015) identified were similar to ostriches. Remnants of ungual sheaths were also present on digits III and IV with an apparent flat wear facet on the fourth (see Fig. 3C in Cuesta et al., 2015). The *Australovenator* podotheca was reconstructed based off comparisons made with both *Dromaius* and *Concavenator*. The plantar surface was reconstructed with small pronounced rounded papillae. Large hexagon like scales cover the cranial portions of each digit, with six scales covering digits III and IV and sixteen cover the cranial surface of digit III. Wear facets were reconstructed on the unguals of digits III and IV whereas digit II was kept relatively sharp. The sheaths were reconstructed based on the average sheath limits dictated in Glen & Bennett (2007) and the underlying bone morphology (Fig. 12). With the estimated soft tissue ROM, internal biology and outer skin developed, the ROM of the pes can be visually synthesised for future foot print creation (Fig. 13).

## Discussion

The analysis of the *Dromaius* pes revealed that overall ROM was greater without the presence of soft tissue. The variation was used as a guide to determine the ROM of the *Australovenator* pes with the presence of soft tissue.

Significant ROM variation was identified with the *Dromaius* unguals. Their proximal ends are osteologically less pronounced with subtle articulation facets with the corresponding phalanx. This morphology resulted in the over estimation of the ungual ROM without soft tissue. This overestimation of ungual ROM is evident in other dinosaurian ROM analyses (including: Carpenter, 2002; Carpenter & Smith, 2001; Galton, 1971; Kobayashi & Barsbold, 2005; Senter, 2005; Senter & Robins, 2005; Senter, 2009). Interestingly Hutson & Hutson (2015) discovered that ROM overestimations of finger and toe joints were the result of rounded concavo/convex articulations while flattened finger and toe joints maybe underestimated due to loss of more rounded cartilage. Subsequently, the bone on bone ROM of the *Australovenator* specimens are overestimates due to their rounded concavo/convex articulations. The ROM of the *Dromaius* pes with and without soft tissue identified that its presence significantly reduces the ROM in the pes.

In spite of this, the most practical method of comparing ROM from an evolutionary perspective is still bone on bone analysis due to the historical bone on bone ROM analysis (Carpenter, 2002; Carpenter & Smith, 2001; Galton, 1971; Kobayashi & Barsbold, 2005; Senter, 2005; Senter & Robins, 2005; Senter, 2009; White et al., 2015a). However, in the future, authors should clarify whether allowances have been made for the presence of soft tissue specifically for accurate evolutionary comparisons.

A pedal ROM comparison with other theropods with known ROM obtained from Senter (2009) did not reveal any significant similarities from an evolutionary standpoint unlike those identified for the *Australovenator* forearm ROM analysis conducted by White et al. (2015b). This is possibly due to the small sample size. However, the ROM data in degrees is presented here to increase the known theropod data set and for future foot print replication analysis (Table 2).

The ROM of pedal phalanx II-3 was obscured by a pathology on its articular facet. The bone is splayed outward as a thin lip, possibly resulting from constant impacts. What caused the pathology is unknown however we speculate that the ungual could have been used as a wounding implement much like the extant ratite *Casuarius casuarius* (Linnaeus, 1758) (commonly known as a cassowary). This bird, along with *Dromaius*, are known to kick during intraspecific fights or when threatened (Davies, 2002; Kofron, 1999; Kofron, 2003). One particular zookeeper described being wounded on two separate occasions by a sandhill crane and an emu. In both cases the impact was initiated with the second digit (Senter, 2009). *Casuarius* are renowned for kicking and interestingly, like *Australovenator*, the largest of the pedal unguals is on the second digit. Consequently, the latter might be a plausible explanation for the existence of the proximal pathology on the ungual.

Interestingly, the dissection of the *Dromaius* pes revealed the extensive mobility of the second toe compared to digits III and IV. This mobility was visualized with the manual manipulation of the FPDII which was not anchored to the metatarsus as were FPDIII and FPDIV. This increased mobility associated with the soft tissue structure and the behavioral aspect of kicking demonstrated in both *Dromaius* and *Casuarius* possibly explains the existence of the pathology on pedal phalanx II-3 of *Australovenator*.

## Conclusions

This study utilised various *in silico* and *ex vivo* techniques to help replicate the biology and ROM of a complete *Australovenator* pes. The soft tissue was reconstructed with a direct comparison of a *Dromaius* pes dissection combined with the overall morphology of the *Australovenator* pes. The phalanges of *Dromaius* were found to have extensive cartilage that extended the ventral and dorsal morphology of the proximal ends of the phalanges. These cartilage extensions, particularly the ventral portion of the phalanx reduced the ROM mostly during flexion. The lowest ROM variation with and without soft tissue was the flexion capabilities of the first phalanx of each digit. Whereas the largest ROM variation was identified with every ungual, with significant reduction in ROM with the presence of soft tissue. Due to the severed tendons caused by butchering, it is unclear how much these potentially increased the ROM. The skin and fatty tissue of the digit pads also reduced the ROM of the *Dromaius* pes.

ROM variation between the presence and absence of soft tissue was used as a guide for hypothesizing a soft tissue ROM for the *Australovenator* pes. This is most likely conservative as this analysis focuses on a severed *Dromaius* tarsometatarsus.

Replication of the skin is also extremely important for future ichnological analysis. The podotheca reconstruction was based on the *Dromaius* pes and the fossilised podotheca of *Concavenator*. The extent of the sheath and their corresponding wear facets, were calculated from known sheath calculations and the existing wear facets on the *Dromaius* unguals.

Traditionally, comparisons of ROM have not allowed for soft tissue. However, as these methods become more advanced the replication of accurate movement can be achieved, which will provide a better understanding of extinct animal behavior.

In the case of *Australovenator*, the ROM of the pes with the allowance for soft tissue has provided the framework for replicating its corresponding footprint. This biological reconstruction will enable comparisons to be made of other suspected theropod footprints found in the local with the replicated prints of *Australovenator*.

##  Supplemental Information

10.7717/peerj.2312/supp-1 Figure S1 Pedal phalanx I-1 Right pedal phalanx in: (A, B) Cranial; (C, D) Ventral; (E, F) Medial; (G, H) Lateral; (I, J) proximal; (K, L) distal.

10.7717/peerj.2312/supp-2 Figure S2 Pedal phalanx I-2 Left pedal phalanx I-2 in: (A, B) Caudal; (C, D) Ventral; (E, F) Medial; (G, H) Lateral; (I, J) Proximal. Right pedal phalanx I-2 in: (K) Caudal; (L) Ventral; (M) Medial; (N) Lateral (O) Proximal.

10.7717/peerj.2312/supp-3 Figure S3 Pedal phalanx II-1 Left pedal phalanx II-1 in: (A, B) Cranial; (C, D) Ventral; (E, F) Ventral; (G, H) Medial; (I, J) Proximal; (K, L) Distal.

10.7717/peerj.2312/supp-4 Figure S4 Pedal phalanx II-2 Left pedal phalanx II-2 in: (A, B) Cranial; (C, D) Ventral; (E, F) Medial (G, H) Lateral; (I, J) Proximal; (K, L) Distal.

10.7717/peerj.2312/supp-5 Figure S5 Pedal phalanx II-3 Left pedal phalanx II-3 in: (A, B) Lateral; (C, D) Cranial; (E, F) Medial; (G, H) Ventral; (I, J) Proximal.

10.7717/peerj.2312/supp-6 Figure S6 Pedal phalanx III-1 Right pedal phalanx III-1 in: [b](A, B) Cranial; (C, D) Ventral; (E, F) Medial; (G, H) Lateral; (I, J) Proximal; (K, L) Distal. Left pedal phalanx in: (M, N) Cranial; (O, P) Ventral; (Q, R) Medial; (S, T) Lateral; (U, V) Proximal; (W, X) distal.

10.7717/peerj.2312/supp-7 Figure S7 Pedal phalanx III-2 Left pedal phalanx III-2 in: (A, B) Caudal; (C, D) Ventral; (E, F) Medial; (G, H) Lateral; (I, J) Proximal; (K, L) Distal.

10.7717/peerj.2312/supp-8 Figure S8 Pedal phalanx III-3 Left pedal phalanx III-3 in: (A, B) Dorsal; (C, D) Medial; (E, F) Ventral; (G, H) Lateral; (I, J) Proximal; (K, L) Distal. Right pedal phalanx III-3 in: (M) Caudal; (N) Ventral; (O) Medial; (P) Lateral; (Q) Proximal; (R) Distal (R).

10.7717/peerj.2312/supp-9 Figure S9 Pedal phalanx III-4 Left pedal phalanx III-4 in: (A, B) Cranial; (C, D) Ventral; (E, F) Medial; (G, H) Lateral; (I, J) Proximal.

10.7717/peerj.2312/supp-10 Figure S10 Pedal phalanx IV-1 Left pedal phalanx IV-1 in: (A, B) Cranial; (C, D) Ventral; (E, F) Medial (G, H) Lateral; (I, J) Proximal; (K, L) Distal.

10.7717/peerj.2312/supp-11 Figure S11 Pedal phalanx IV-2 Left pedal phalanx IV-2 in: (A, B) Cranial; (C, D) Ventral; (E, F) Medial; (G, H) Lateral; (I, J) Proximal; (K, L) Distal.

10.7717/peerj.2312/supp-12 Figure S12 Pedal phalanx IV-3 Left pedal phalanx IV-3 in: (A, B) Cranial; (C, D) Ventral; (E, F) Medial; (G, H) Lateral; (I, J) Proximal; (K, L).

10.7717/peerj.2312/supp-13 Figure S13 Pedal phalanx IV-4 Left pedal phalanx IV-4 in: (A, B) Cranial; (C, D) Ventral; (E, F) Medial; (G, H) Lateral; (I, J) Proximal; (K, L) Distal.

10.7717/peerj.2312/supp-14 Figure S14 Pedal phalanx IV-5 Right pedal phalanx IV-5 in: (A, B) Cranial; (C, D) Ventral; (E, F) Medial (G, H) Lateral; (I, J) Proximal.

10.7717/peerj.2312/supp-15 Table S1 Pedal phalanx I-1 measurements * Best estimate due to poor preservation. Measurements in mm.

## Additional Information and Declarations

### Field Study Permissions

The following information was supplied relating to field study approvals (i.e., approving body and any reference numbers): Permission to excavate the specimens from Elderslie station was obtained from the landholders who donate all specimens to the Australian Age of Dinosaur Museum of Natural History (AAOD).

### Data Availability

The following information was supplied regarding data availability: The raw data has been supplied as Supplemental Dataset.
