# Supplementary figures and images for: The pes of Australovenator wintonensis (Theropoda: Megaraptoridae): analysis of the pedal range of motion and biological restoration
Source: PeerJ. 2016 Aug 3;4:e2312. doi: 10.7717/peerj.2312 (PMC4975041; doi:10.7717/peerj.2312)

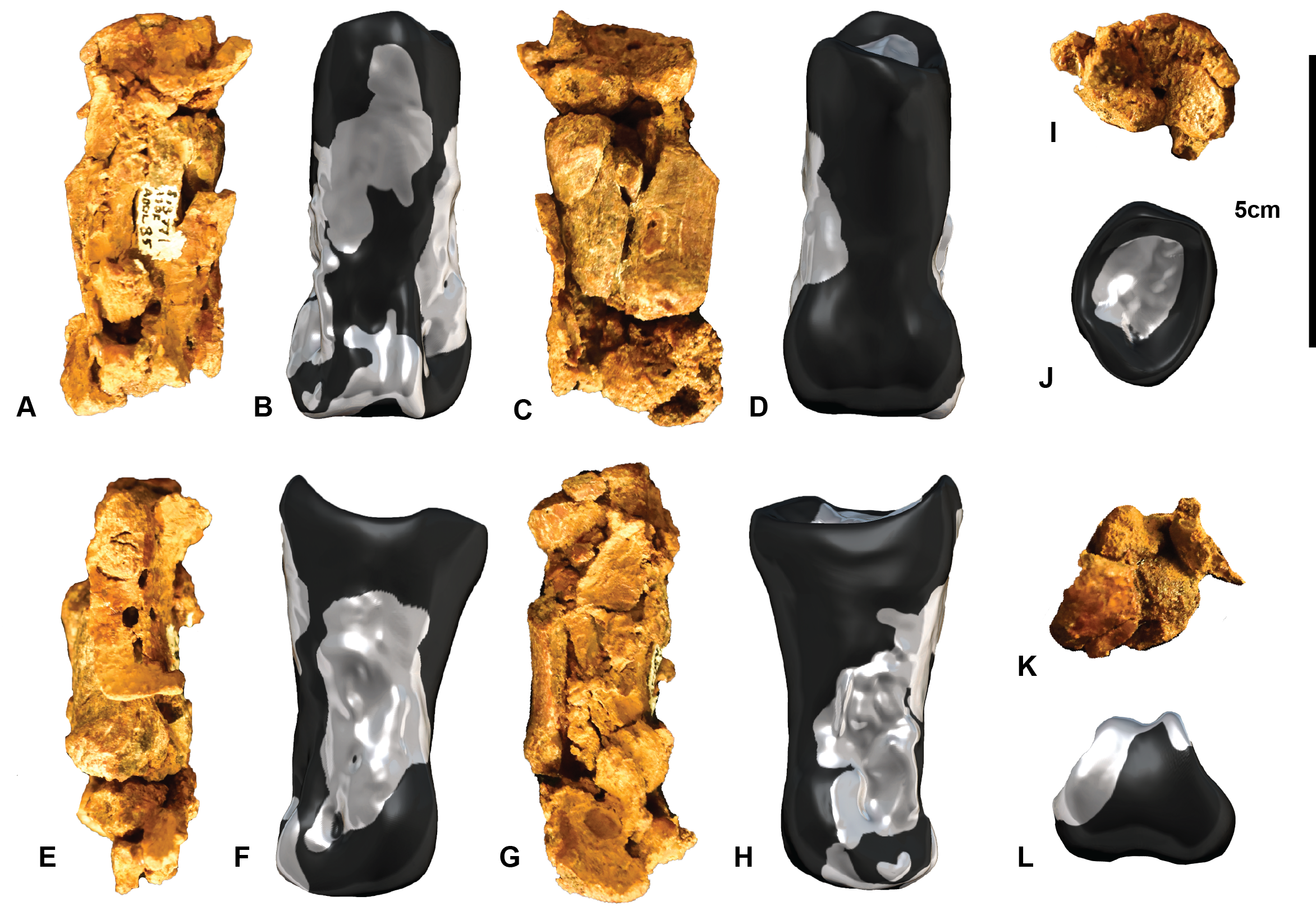

Supplement: Figure S1 — Right pedal phalanx in: (A, B) Cranial; (C, D) Ventral; (E, F) Medial; (G, H) Lateral; (I, J) proximal; (K, L) distal. [file peerj-04-2312-s001.png]

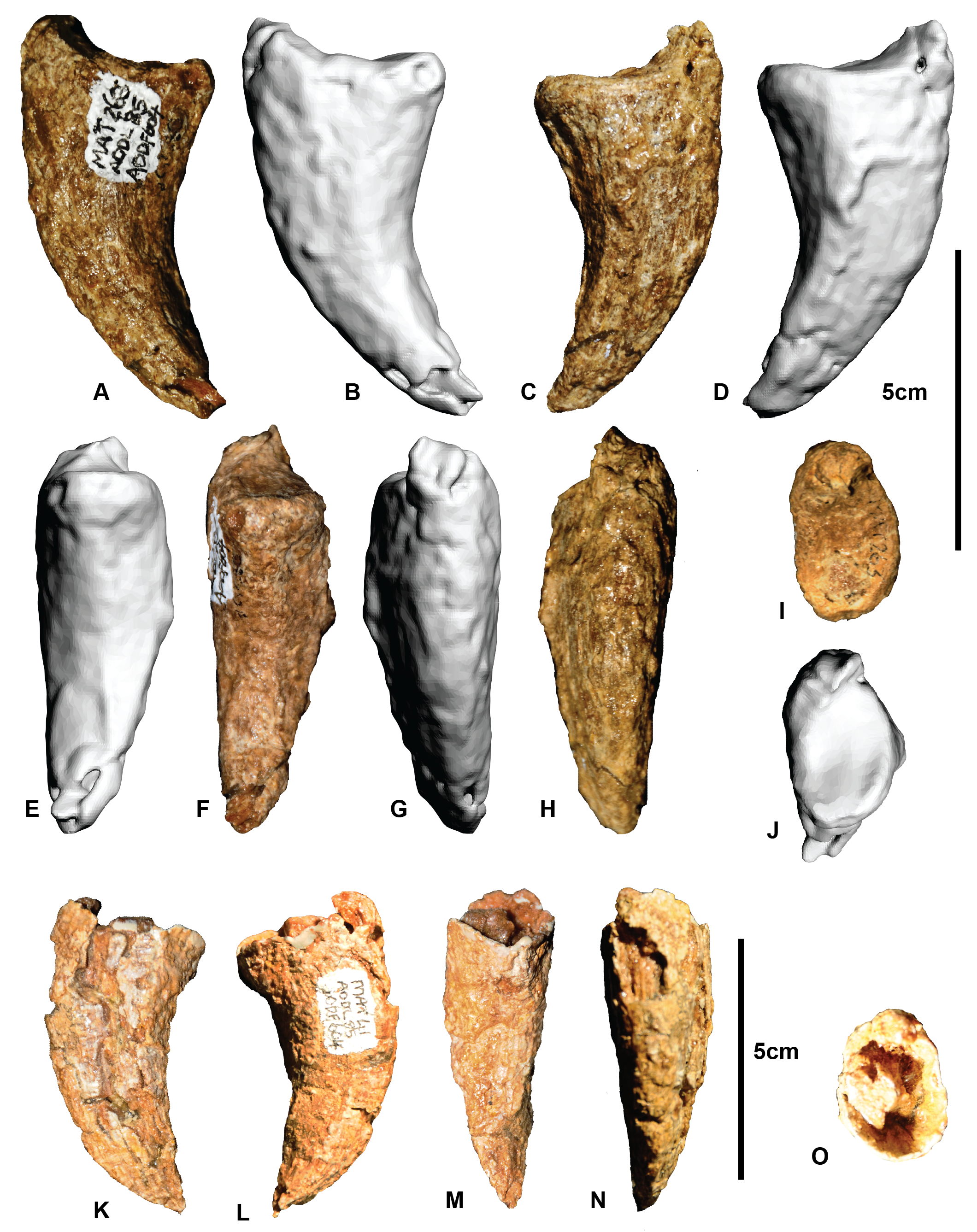

Supplement: Figure S2 — Left pedal phalanx I-2 in: (A, B) Caudal; (C, D) Ventral; (E, F) Medial; (G, H) Lateral; (I, J) Proximal. Right pedal phalanx I-2 in: (K) Caudal; (L) Ventral; (M) Medial; (N) Lateral (O) Proximal. [file peerj-04-2312-s002.png]

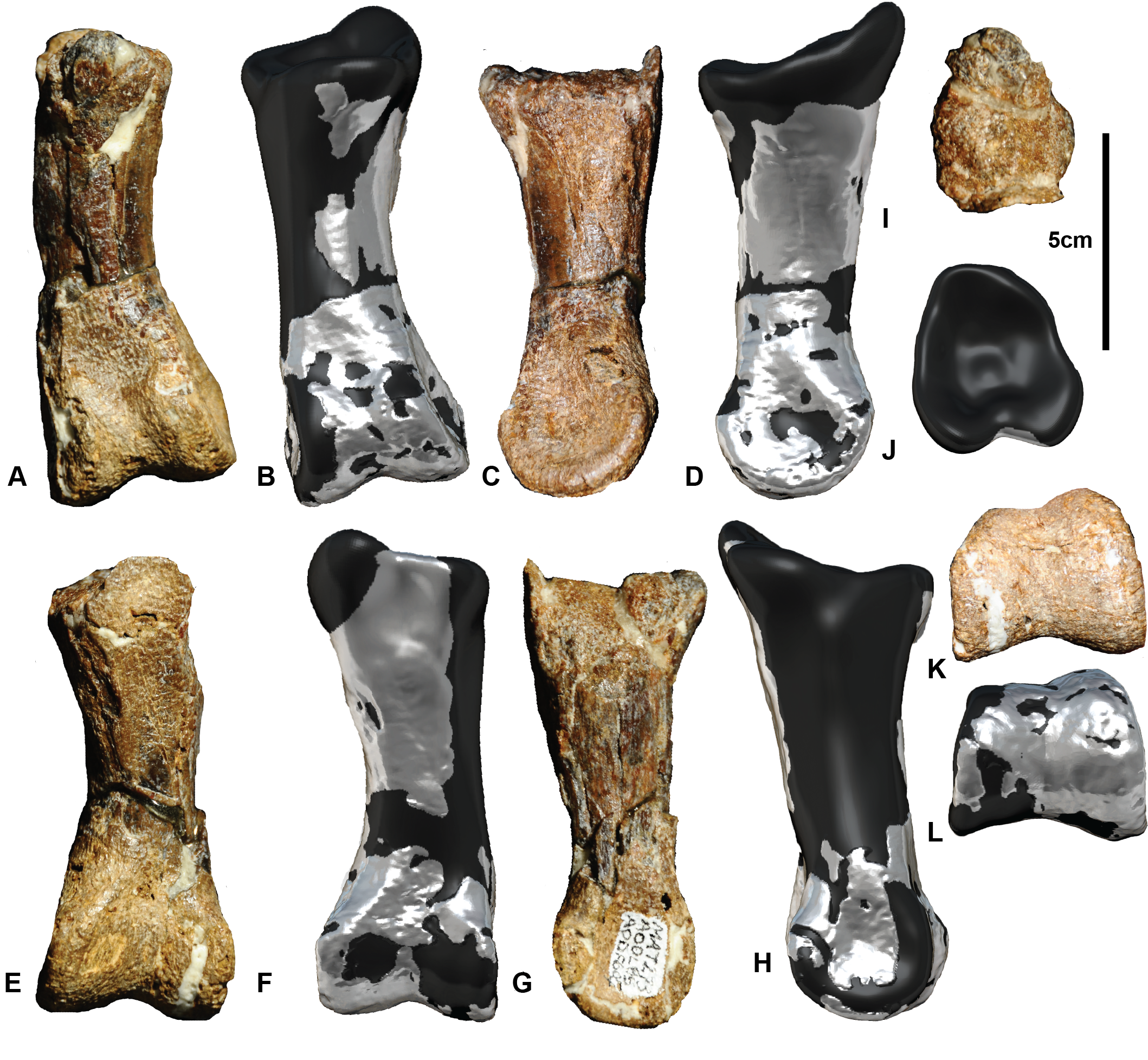

Supplement: Figure S3 — Left pedal phalanx II-1 in: (A, B) Cranial; (C, D) Ventral; (E, F) Ventral; (G, H) Medial; (I, J) Proximal; (K, L) Distal. [file peerj-04-2312-s003.png]

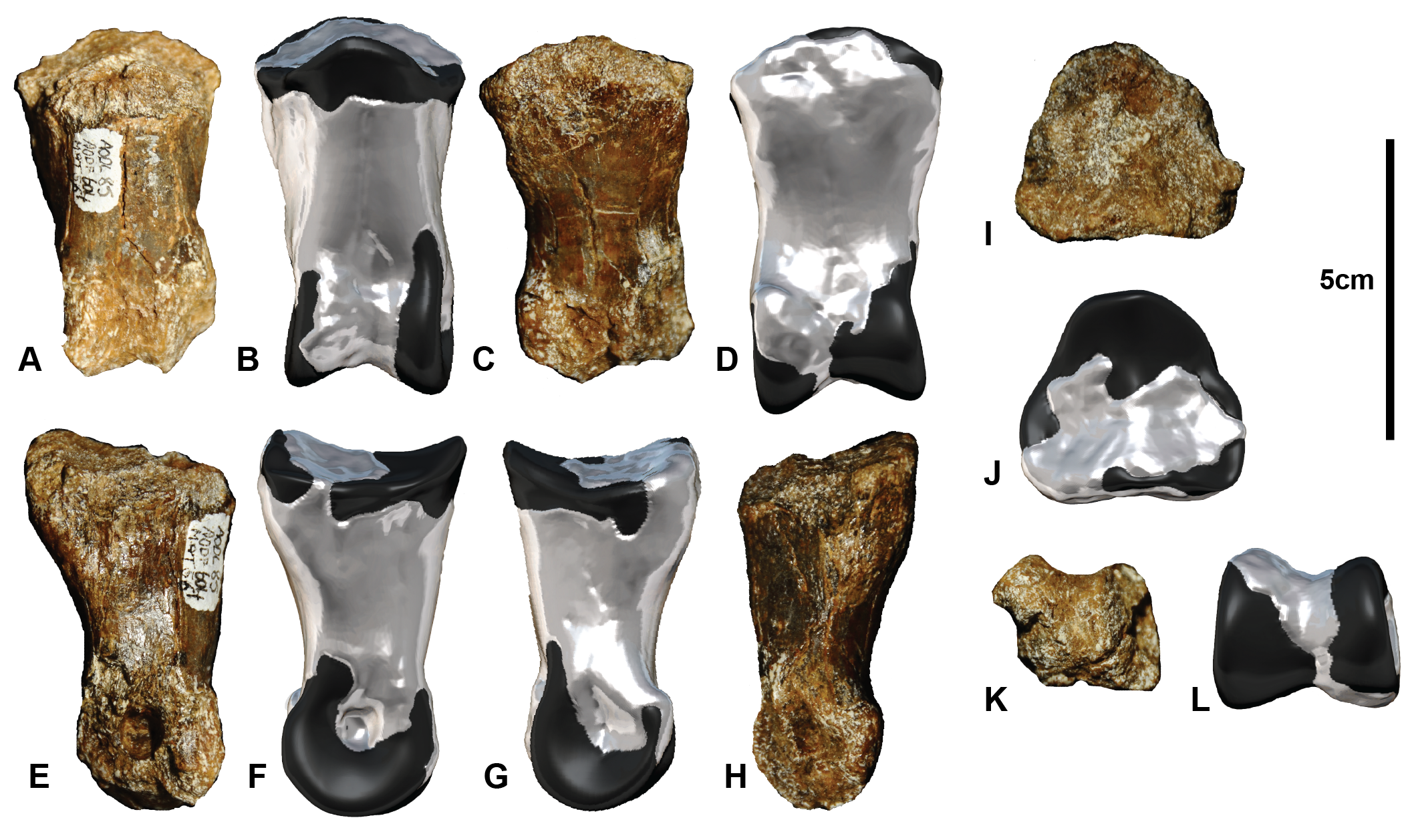

Supplement: Figure S4 — Left pedal phalanx II-2 in: (A, B) Cranial; (C, D) Ventral; (E, F) Medial (G, H) Lateral; (I, J) Proximal; (K, L) Distal. [file peerj-04-2312-s004.png]

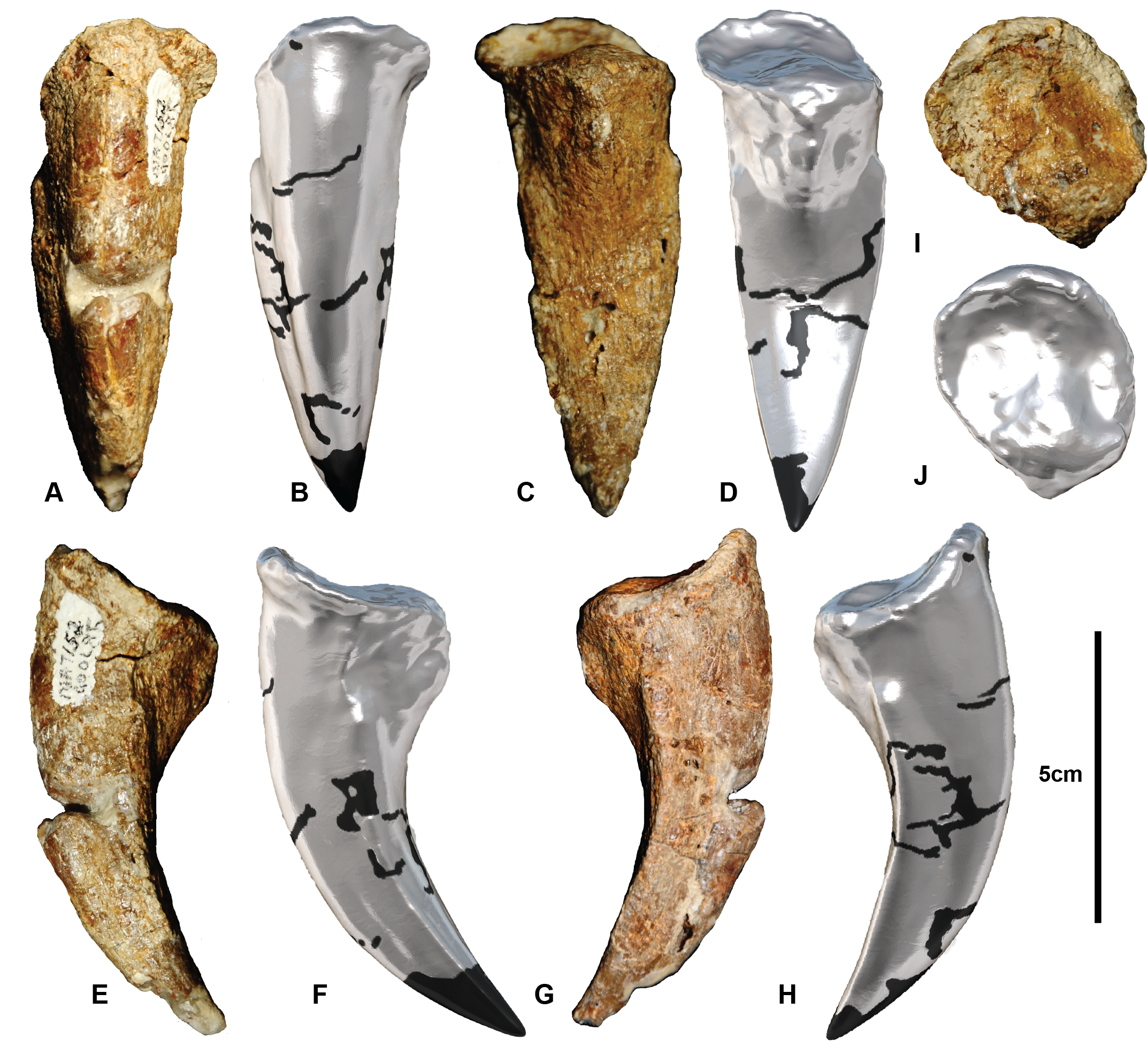

Supplement: Figure S5 — Left pedal phalanx II-3 in: (A, B) Lateral; (C, D) Cranial; (E, F) Medial; (G, H) Ventral; (I, J) Proximal. [file peerj-04-2312-s005.png]

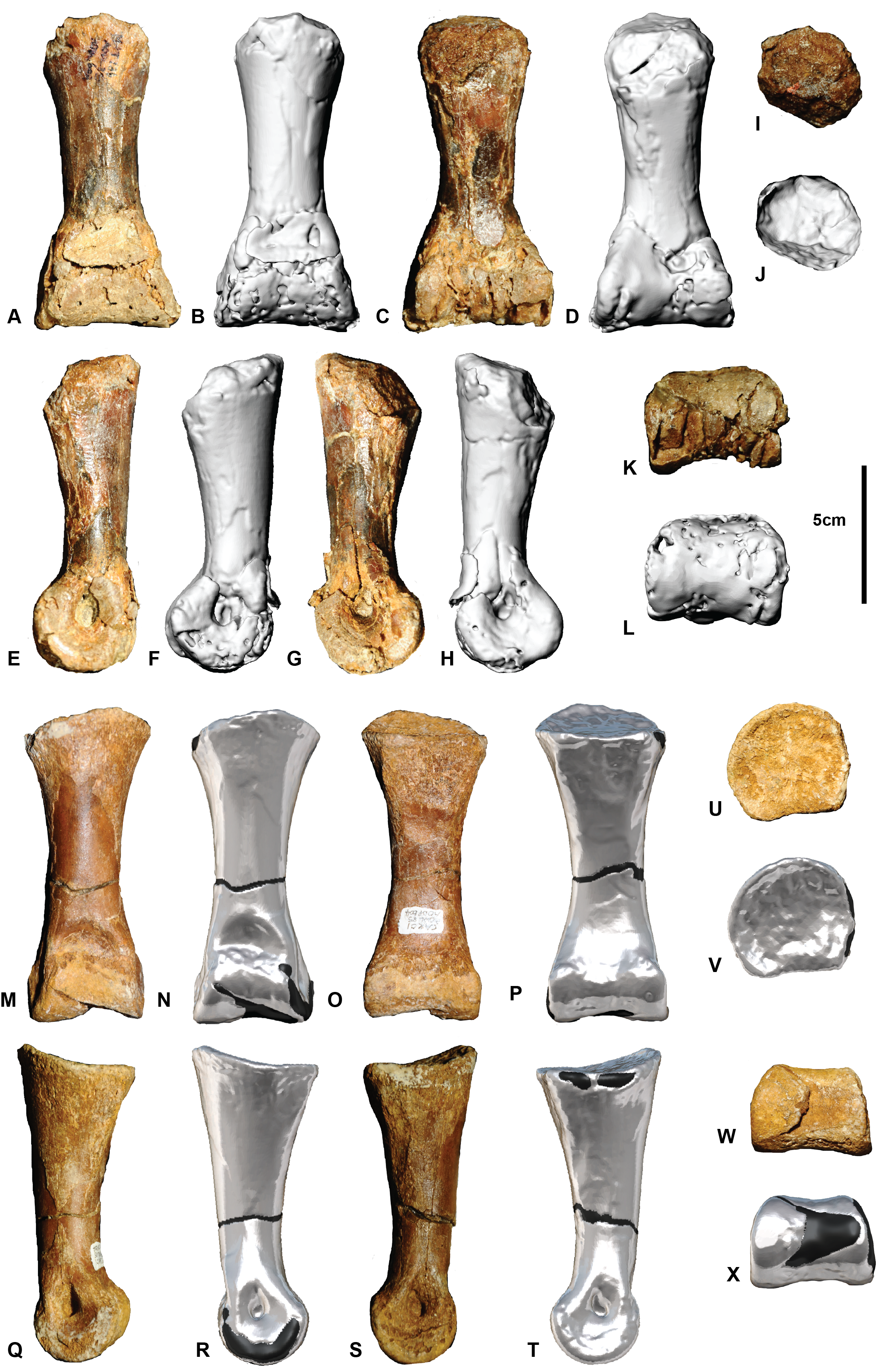

Supplement: Figure S6 — Right pedal phalanx III-1 in: [b](A, B) Cranial; (C, D) Ventral; (E, F) Medial; (G, H) Lateral; (I, J) Proximal; (K, L) Distal. Left pedal phalanx in: (M, N) Cranial; (O, P) Ventral; (Q, R) Medial; (S, T) Lateral; (U, V) Proximal; (W, X) distal. [file peerj-04-2312-s006.png]

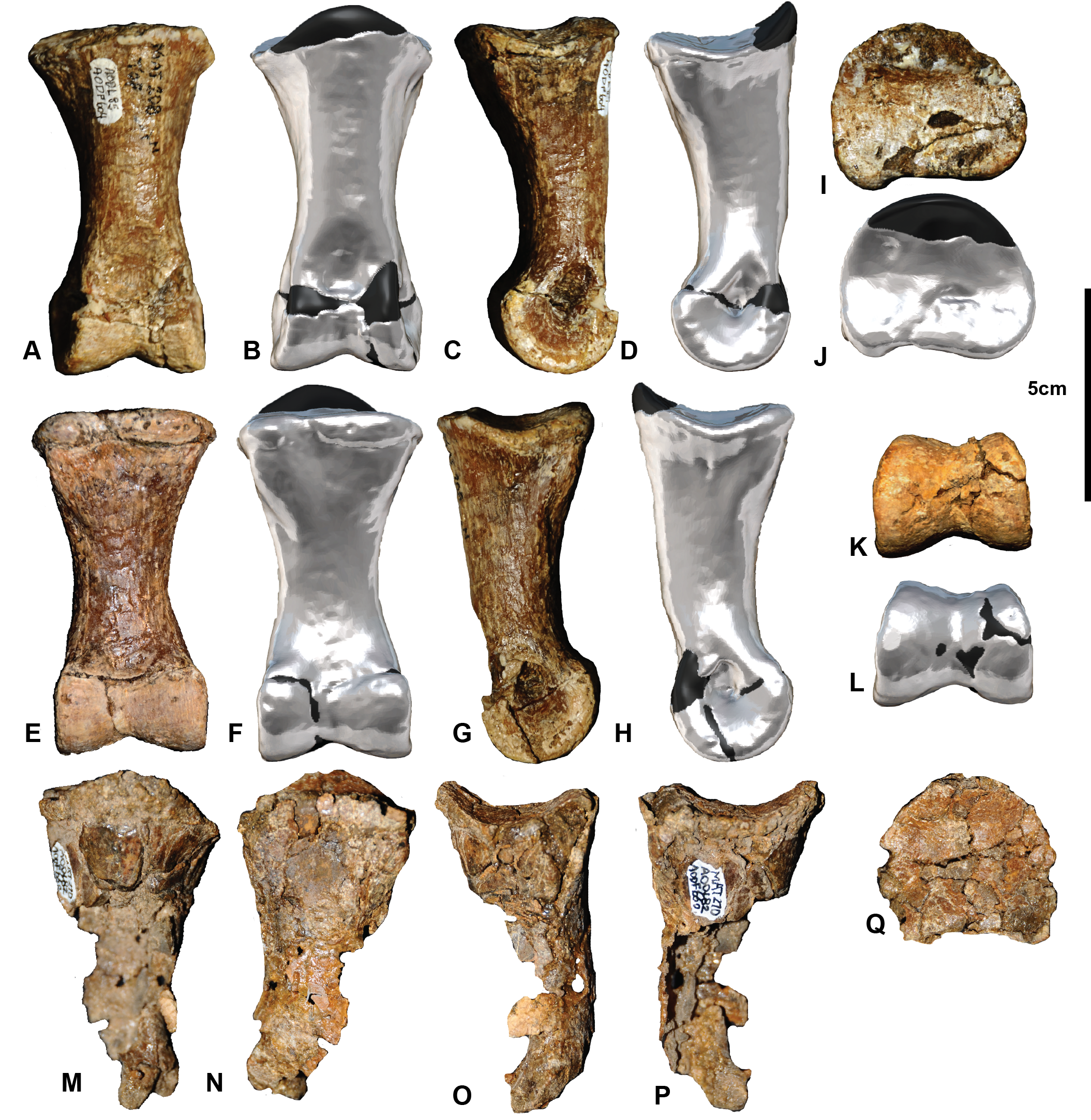

Supplement: Figure S7 — Left pedal phalanx III-2 in: (A, B) Caudal; (C, D) Ventral; (E, F) Medial; (G, H) Lateral; (I, J) Proximal; (K, L) Distal. [file peerj-04-2312-s007.png]

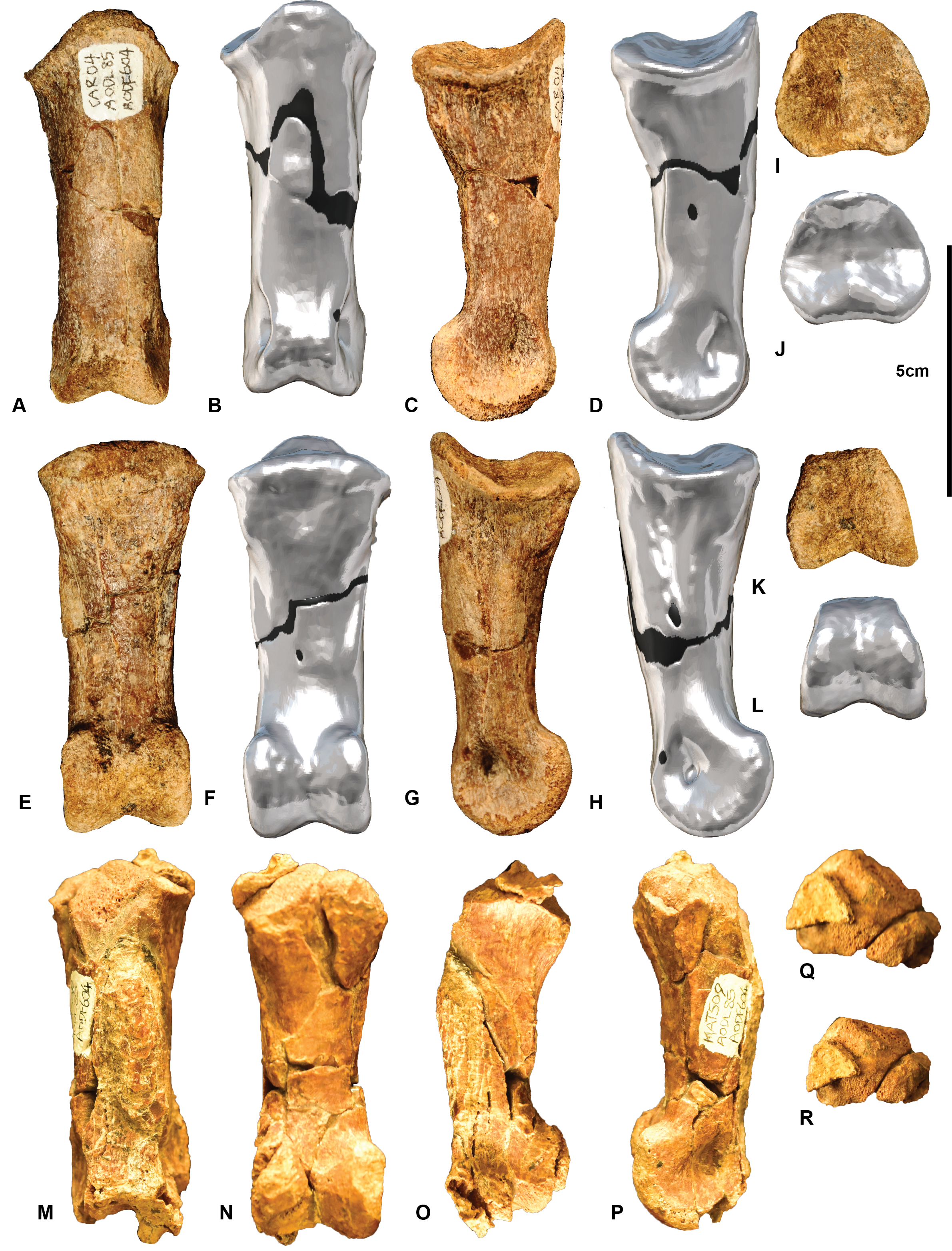

Supplement: Figure S8 — Left pedal phalanx III-3 in: (A, B) Dorsal; (C, D) Medial; (E, F) Ventral; (G, H) Lateral; (I, J) Proximal; (K, L) Distal. Right pedal phalanx III-3 in: (M) Caudal; (N) Ventral; (O) Medial; (P) Lateral; (Q) Proximal; (R) Distal (R). [file peerj-04-2312-s008.png]

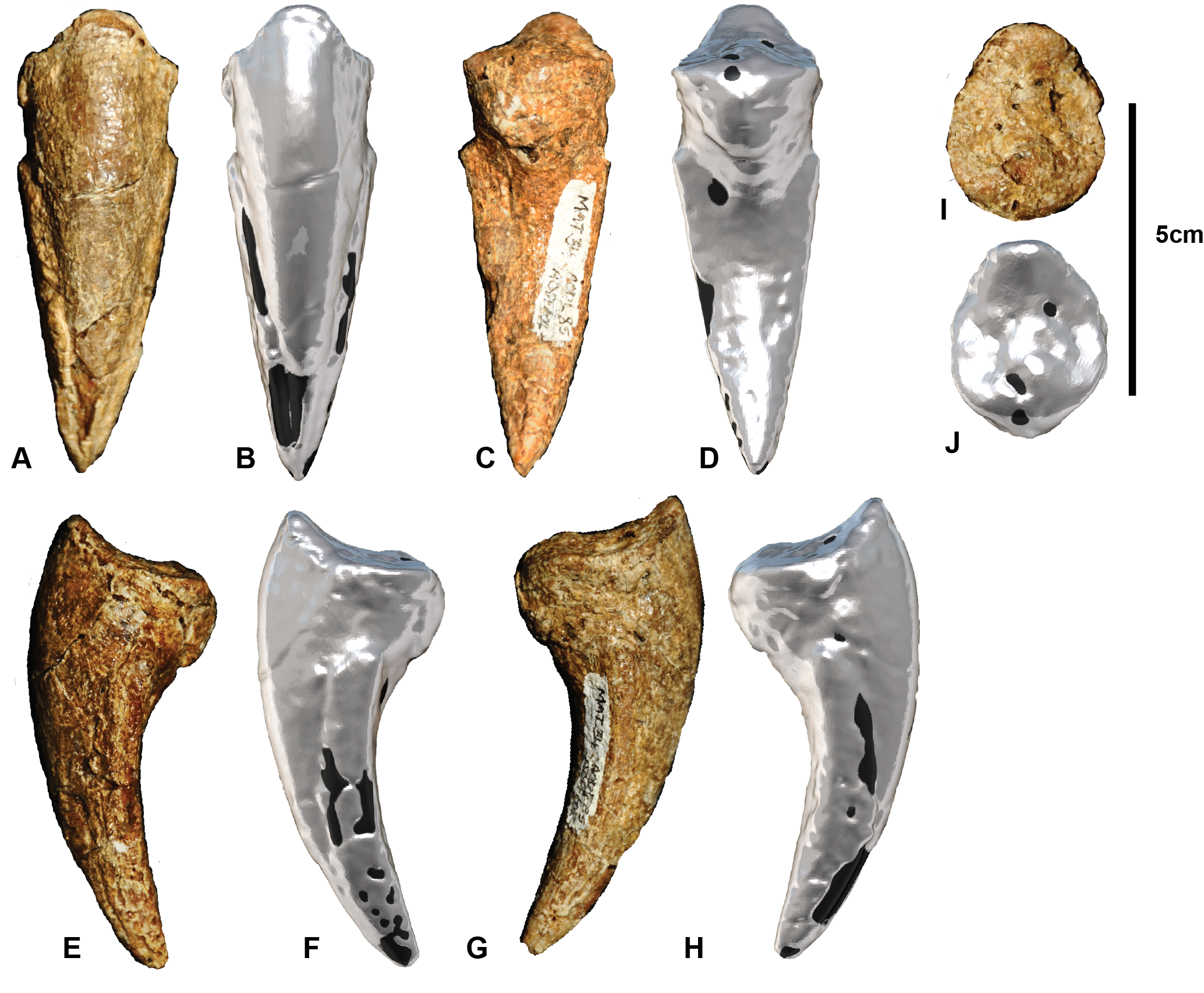

Supplement: Figure S9 — Left pedal phalanx III-4 in: (A, B) Cranial; (C, D) Ventral; (E, F) Medial; (G, H) Lateral; (I, J) Proximal. [file peerj-04-2312-s009.png]

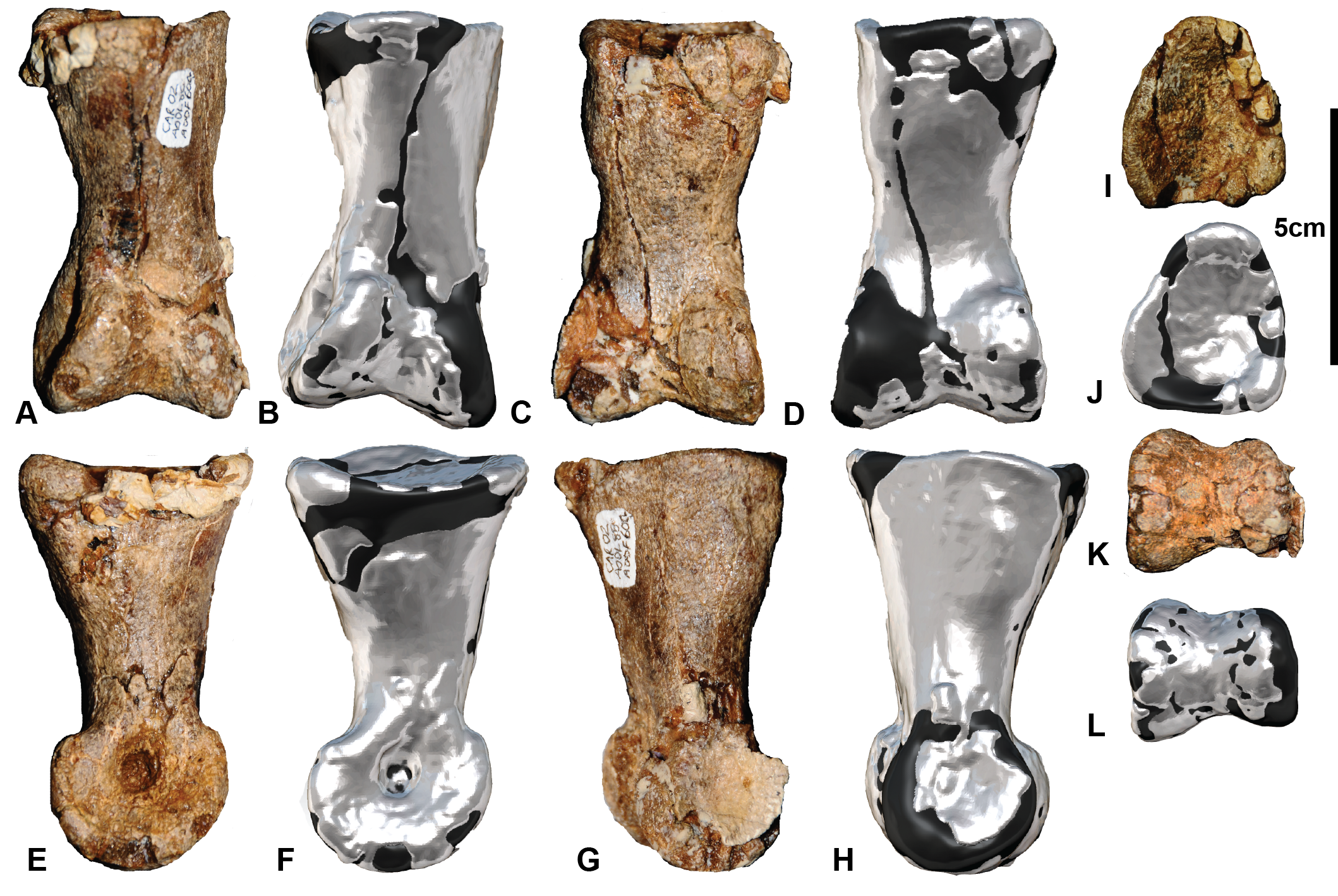

Supplement: Figure S10 — Left pedal phalanx IV-1 in: (A, B) Cranial; (C, D) Ventral; (E, F) Medial (G, H) Lateral; (I, J) Proximal; (K, L) Distal. [file peerj-04-2312-s010.png]

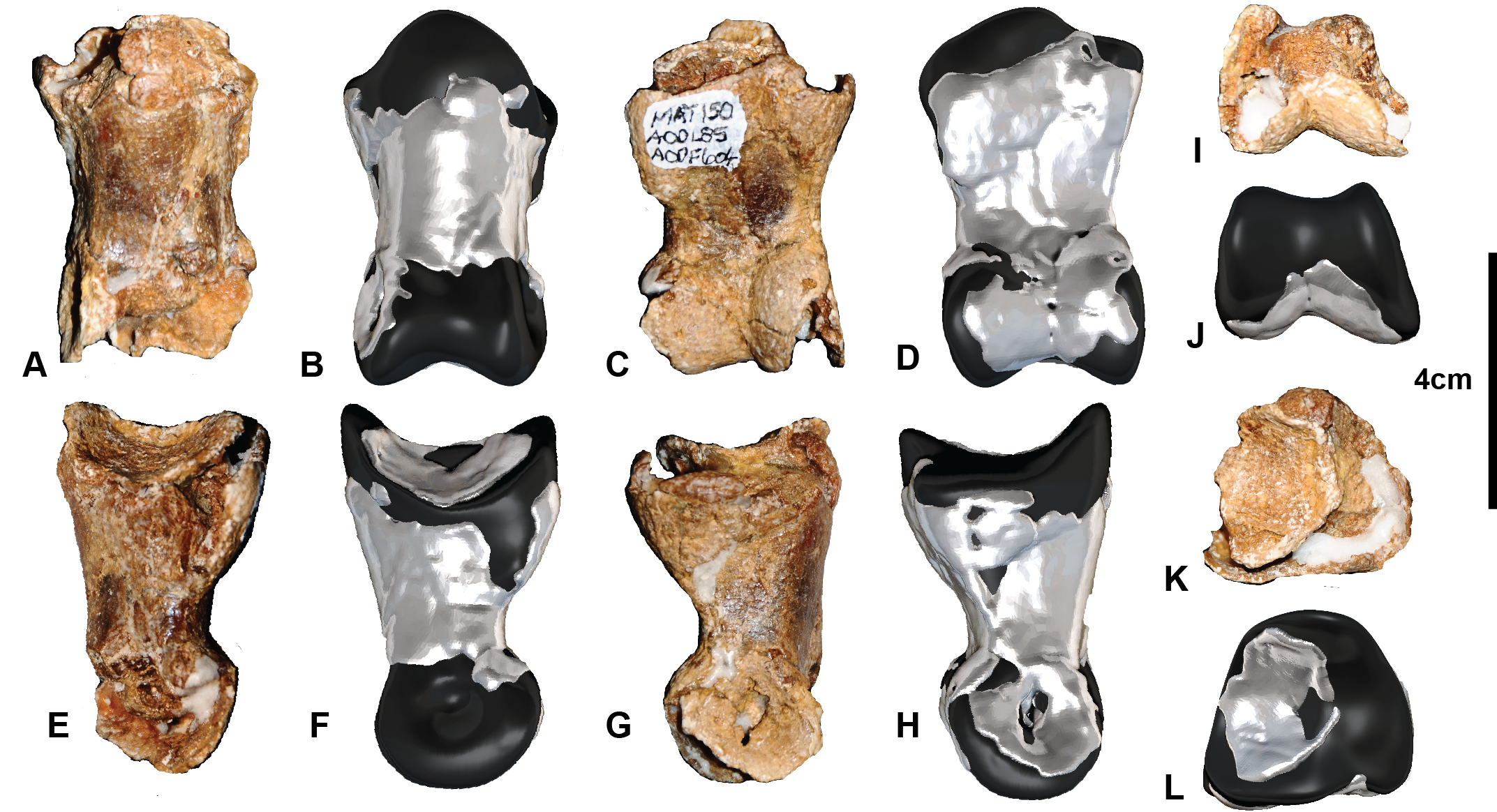

Supplement: Figure S11 — Left pedal phalanx IV-2 in: (A, B) Cranial; (C, D) Ventral; (E, F) Medial; (G, H) Lateral; (I, J) Proximal; (K, L) Distal. [file peerj-04-2312-s011.png]

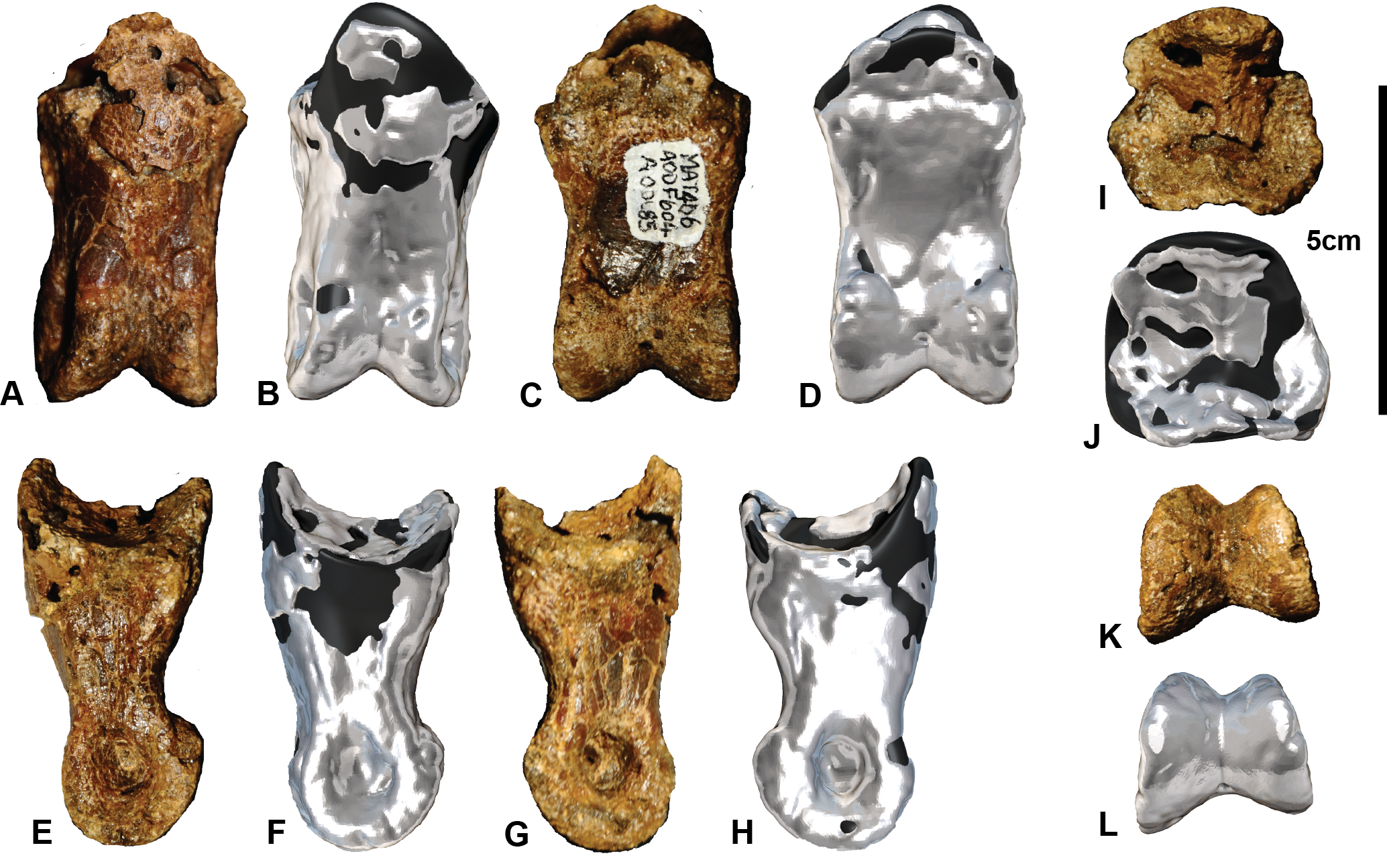

Supplement: Figure S12 — Left pedal phalanx IV-3 in: (A, B) Cranial; (C, D) Ventral; (E, F) Medial; (G, H) Lateral; (I, J) Proximal; (K, L). [file peerj-04-2312-s012.png]

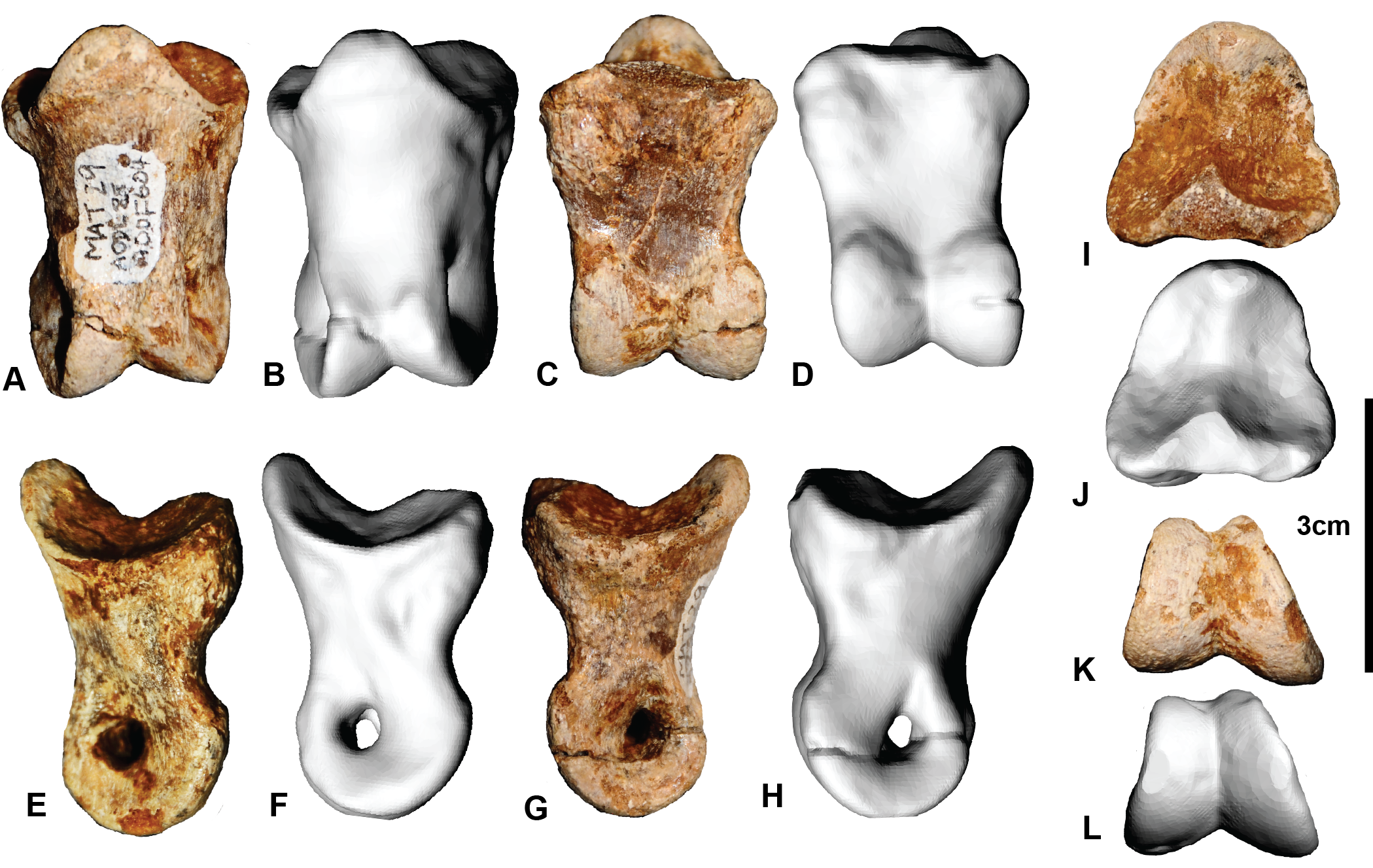

Supplement: Figure S13 — Left pedal phalanx IV-4 in: (A, B) Cranial; (C, D) Ventral; (E, F) Medial; (G, H) Lateral; (I, J) Proximal; (K, L) Distal. [file peerj-04-2312-s013.png]

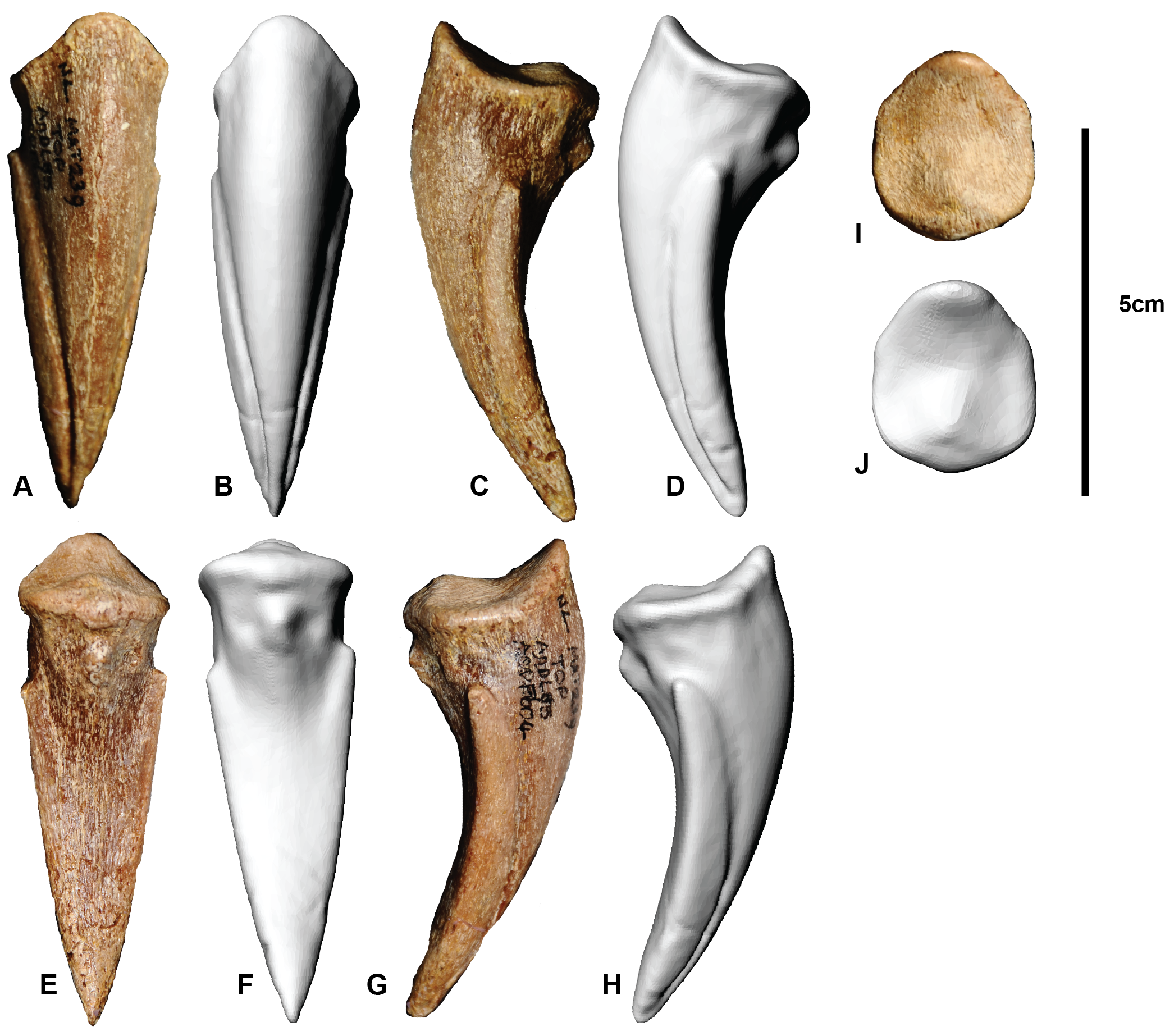

Supplement: Figure S14 — Right pedal phalanx IV-5 in: (A, B) Cranial; (C, D) Ventral; (E, F) Medial (G, H) Lateral; (I, J) Proximal. [file peerj-04-2312-s014.png]
