# Supplementary material for: The pes of Australovenator wintonensis (Theropoda: Megaraptoridae): analysis of the pedal range of motion and biological restoration
Source: PeerJ. 2016 Aug 3;4:e2312. doi: 10.7717/peerj.2312 (PMC4975041; doi:10.7717/peerj.2312)
Supplement: Table S1 — * Best estimate due to poor preservation. Measurements in mm. [file peerj-04-2312-s015.docx]

Table S1: Specimen measurements of pedal phalanx I-1. * Best estimate due to poor preservation. Measurements in mm.

| Length | 66 |
| --- | --- |
| Proximal height | 20* |
| Proximal width | 30* |
| Distal height | 22* |
| Distal width | 25* |
